# Automatic Annotation of Spatial Expression Patterns via Sparse Bayesian Factor Models

**DOI:** 10.1371/journal.pcbi.1002098

**Published:** 2011-07-21

**Authors:** Iulian Pruteanu-Malinici, Daniel L. Mace, Uwe Ohler

**Affiliations:** 1 Institute for Genome Sciences and Policy, Duke University, Durham, North Carolina, United States of America; 2 University of Washington, Seattle, Washington, United States of America; Johns Hopkins University, United States of America

## Abstract

Advances in reporters for gene expression have made it possible to document and quantify expression patterns in 2D–4D. In contrast to microarrays, which provide data for many genes but averaged and/or at low resolution, images reveal the high spatial dynamics of gene expression. Developing computational methods to compare, annotate, and model gene expression based on images is imperative, considering that available data are rapidly increasing. We have developed a sparse Bayesian factor analysis model in which the observed expression diversity of among a large set of high-dimensional images is modeled by a small number of hidden common factors. We apply this approach on embryonic expression patterns from a *Drosophila* RNA *in situ* image database, and show that the automatically inferred factors provide for a meaningful decomposition and represent common co-regulation or biological functions. The low-dimensional set of factor mixing weights is further used as features by a classifier to annotate expression patterns with functional categories. On human-curated annotations, our sparse approach reaches similar or better classification of expression patterns at different developmental stages, when compared to other automatic image annotation methods using thousands of hard-to-interpret features. Our study therefore outlines a general framework for large microscopy data sets, in which both the generative model itself, as well as its application for analysis tasks such as automated annotation, can provide insight into biological questions.

## Author Summary

High throughput image acquisition is a quickly increasing new source of data for problems in computational biology, such as phenotypic screens. Given the very diverse nature of imaging technology, samples, and biological questions, approaches are oftentimes very tailored and ad hoc to a specific data set. In particular, the image-based genome scale profiling of gene expression patterns via approaches like *in situ* hybridization requires the development of accurate and automatic image analysis systems for understanding regulatory networks and development of multicellular organisms. Here, we present a computational method for automated annotation of *Drosophila* gene expression images. This framework allows us to extract, identify and compare spatial expression patterns, of essence for higher organisms. Based on a sparse feature extraction technique, we successfully cluster and annotate expression patterns with high reliability, and show that the model represents a “vocabulary” of basic patterns reflecting common function or regulation.

## Introduction

Detailed knowledge of the precise location and time span of gene expression is mandatory to deciphering dynamic cellular mechanisms. The application of microarray technology has led to genome-wide quantitative overviews of the relative changes of transcript levels in many organisms (such as *Drosophila* embryonic development [1]–[4]), but these rarely provide spatial information. In contrast, microscopy of colored or fluorescent probes, followed by imaging, is able to deliver spatial quantitative phenotype information such as gene expression at high resolution [5], [6]. For instance, RNA *in situ* hybridization localizes specific mRNA sequences by hybridizing complementary mRNA-binding oligonucleotides and a suitable dye [7]. This approach has been used as part of large scale compendia of gene expression in *Drosophila* embryos [4], and the adult mouse brain [9], [10].

Available image data therefore constitute a repertoire of distinctive spatial expression patterns, allowing us to obtain significant insights on gene regulation during development or in complex organs. One of the fastest growing expression pattern data collections is the Berkeley *Drosophila* Genome Project RNA *in situ* hybridization database [8]), which contains annotations of spatial expression patterns using a controlled vocabulary, following the example of the Gene Ontology (GO) [11]. The annotation terms integrate the spatial gene expression dimensions of a developing “path” from the cellular blastoderm stage until organs are formed. Over 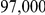 images for 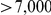 genes have thus been manually acquired, curated and annotated [4]. Due to the complex nature of the task, these *Drosophila* images were manually annotated by human experts.

Automatic image annotation systems are fairly routinely used in cell-based assays, *e.g.* for the classification of protein subcellular localization in budding yeast [12]. The increasing number of expression images for complex organisms has motivated the design of computational methods to automate these analyses [13]. In general, this requires solving two sub-problems: identifying objects in a potentially noisy image and normalizing the morphology of the objects, followed by analysis on the actual expression patterns. Typically, studies have focused on the task to recapitulate the expert-provided annotation, based on bottom-up approaches utilizing large sets of low-level features extracted from the images. For instance, Ji *et al.* [14] proposed a bag-of-words scheme in which invariant visual features were first extracted from local patches on the images, followed by a feature quantization based on precomputed “visual codebooks” and finally classification. Peng *et al.* [15] developed an automatic image annotation framework using three different feature representations (based on Gaussian Mixture Models, Principal Component Analysis (PCA) and wavelet functions) and several classifiers, including Linear Discriminant Analysis (LDA), Support Vector Machines (SVM) and Quadratic Discriminant Analysis (QDA). Heffel *et al.* [16] proposed an embryo outline extraction and transformation and conversion to Fourier coefficients-based feature representation.

One potential drawback of the above mentioned approaches is the high dimensional and complex feature space (thousands of features per image) which implies a potential for high redundancy and computational difficulties. In contrast to such large feature sets, a spatial expression pattern typically consists of a limited number of discrete domains, defined by a small set of upstream regulatory factors. As an alternative, Frise *et al.* [17] therefore set out to identify a concise set of basic expression patterns in *Drosophila*. Starting with an unsupervised clustering approach on a manually selected small set of 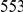 distinct images, the clusters were extended to a broader data set comprising 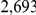 lateral views of early development through a binary SVM classification. This pipeline revealed a set of 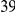 well defined clusters describing specific regions of expression with good correspondence to developmental structures and shared biological functions of the genes within clusters. While the authors gave many individual examples for the possible meaning of clusters, they did not use them in further applications to annotate patterns or infer regulatory relationships. As with most of the described approaches, the study involved significant human intervention, which generally includes manual selection of “good” images for training, clustering, and/or evaluation: selection of a subset of viewpoints (images show different embryo orientations, *e.g.* lateral or dorsal/ventral), or selection of successfully registered images only. While this may lead to highly encouraging results, the significant work for manual image selection represents a potential shortcoming, considering that available data are rapidly increasing and an automatic computational method is essential.

We here propose a new approach to close the gap between the feature-oriented approaches for pattern annotation, and the identification of expression domains to gain functional insights. The central part is the application of sparse Bayesian factor analysis (sBFA), which describes a large number of observed variables (image features) by linear combinations of a much smaller number of unobserved variables (factors). This framework aims at explaining the variability of the original high dimensional feature space by a much smaller set of latent factors, through a completely unsupervised process. [Note that the mathematical usage of the word “factor” is distinct from its biological meaning]. It can also be seen as a clustering method, where samples belong to different clusters, based on their corresponding linear combination mixing weights. Another advantage of the sBFA model is that any information about the underlying structure can be easily incorporated through priors [18]; for instance, we here use a sparseness prior placed on the number of factors used to “reconstruct” each image.

Using such sparse Bayesian approaches we identify a basic expression vocabulary directly from the image data, and show that this small subset of features is highly interpretable in terms of biological function or co-regulation. This vocabulary is then used for gene annotation with performance comparable or exceeding current systems, and stability when applied on the complete and noisy data set, without any human intervention or selection of “representative” images. The top-down generative nature of this approach (rather than traditional bottom-up approaches) also promises high utility in other application areas, by integrating the model with various information on gene expression and regulation.

## Results

Our study describes the application of an sBFA framework for gene expression pattern annotation. The model converts every segmented image of a *Drosophila* embryo into a sparse feature representation of the spatial gene expression pattern, suitable for downstream quantitative analysis based on widely used classifiers. This technique is fully automatic, and not specific to any data or feature set. In the analysis presented here, we employ factor models where data (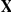) are modeled by a linear combination of factors (rows of 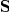) given the corresponding mixing weights (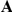) and some additive Gaussian noise (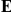) while sparseness is promoted on the factor loading matrix (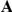). The model jointly infers both the factors and the mixing weights; we can then analyze the factors regarding their representation of biological concepts, and use the mixing weights for analysis tasks such as automatic annotation.

### *Drosophila* image data sets

One of the most popular data sets to explore the use of image expression data is the Berkeley *Drosophila* Genome Project (BDGP) data set. It consists of over 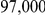 images which document embryonic expression patterns for over 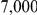 of the 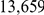 protein-coding genes identified in the *Drosophila melanogaster* genome. A gene's expression pattern can be reflected in the accumulation of its product in subsets of cells as embryonic development progresses. In this case, the patterns of mRNA expression were studied by RNA *in situ* hybridization, which has the potential to reveal the spatial aspects of gene expression during development at genome-wide scale. The RNA *in situ* hybridization used digoxygenin-labeled RNA probes derived primarily from sequenced cDNAs to visualize gene expression patterns and documented them by digital microscopy. For each expressed gene, representative low and high magnification images were captured at key developmental stages. These developmental stages clearly define emerging embryonic structures such as gastrulation, midblastula transition and organogenesis onset. For practical reasons, the first 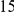 hours of *Drosophila* development, spanning embryonic stages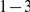, 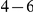, 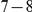, 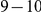, 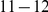 and 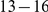, were chosen for analysis, as this interval is manageable in terms of data annotation. As examples, stages 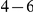 are associated with the time interval 1h20min–3h, while the later developmental stages 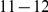 occur between 5h20min–9h20min.

Genes are annotated with ontology terms from a controlled vocabulary describing developmental expression patterns (*cf.* [11]). Any gene is thus associated to one or multiple terms, and often represented by more than one image. Images can display non-informative patterns due to poor quality staining/washing, and a gene can show distinct and different expression patterns due to different embryo orientations or the relatively long developmental time spanned by a stage range. Images with lateral orientation have now been annotated as such, information not available until recently.

As proof of concept, the model is demonstrated on a variety of images, covering two distinct developmental stage ranges (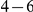, 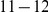) and multiple orientations (lateral, dorsal/ventral). The first data set (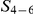) includes 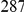 genes (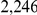 images) with arbitrary orientation (mostly lateral and dorsal/ventral), acquired during the time window of developmental stages 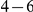. The second data set (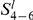) covers a subset of 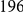 genes (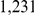 images) restricted to lateral views; we used this smaller data set to evaluate the effect of integrating images from multiple views, and to be able to compare against earlier approaches which were frequently applied on lateral views only. Genes in these two sets were annotated with 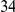 non-trivial terms (i.e. excluding no or ubiquitous expression). The third data set (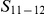) covers 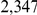 genes (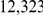 images) with arbitrary orientation from the later developmental stage range 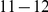. At this point, the problem is complicated by the more developed embryo morphology, which gives rise to intricate spatial expression patterns. Consequently, genes in this set were annotated with 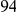 unique non-trivial terms. The last data set (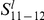) contains 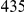 manually selected genes from data set 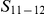 as used in a previous study [15], comprising 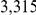 images with lateral view only.

The image registration process used throughout this paper was previously introduced by Mace *et al.* [19] in which individual embryos were extracted and rotated in an automatic fashion. We then scaled the registered images to 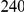
×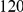 pixel resolution and extracted grid-based features by calculating the mean pixel value within each patch. Details can be found in the “Materials and Methods” section.

### Factor inference and decomposition of expression patterns

**Figure 1:**
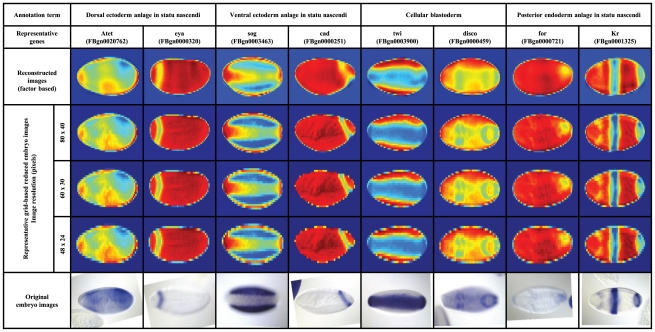
Original, grid-based and reconstructed factor-based images, using the estimated factors and factor loading matrix. Selected annotation terms with the highest number of associated genes; each annotation term is represented by two of its corresponding genes (with the original, the grid-based factor-based embryo images), from the time window of developmental stages 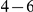. These examples reveal that images with the same annotation term can show different orientations and quite different patterns, for instance because they are taken during a relatively large temporal window during which expression can change. In the false color display, blue color indicates strong *in situ* staining while red indicates no staining.

To illustrate the potential of a sparse set of factors to represent complex expression patterns, we started with data set 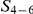. We evaluated different values for the number of factors in the model (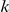) and different resolution – 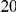, 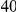 and 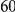 factors for grid sizes of 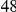
×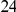, 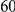
×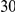 and 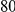
×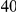, respectively. Representative images (original, grid-based, and reconstructed factor-based) for the annotation terms with the highest number of associated genes are shown in Figure 1. While the resulting images are somewhat noisier, they clearly recapitulate the overall expression domains.

**Figure 2:**
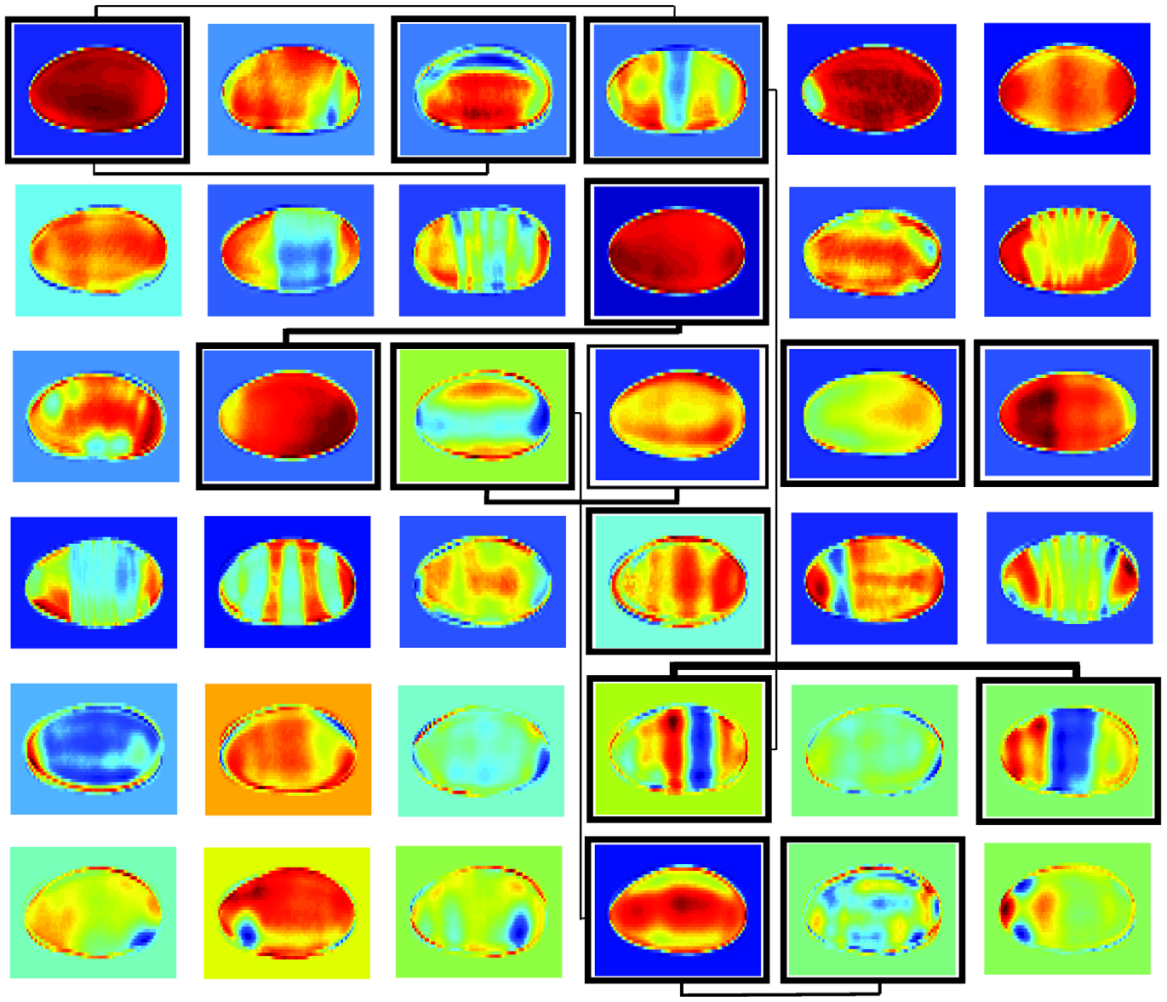
Selected factors estimated from a total of 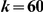 factors, for a grid size of 80×40 (data set 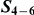. As factors can have negative loadings, patterns may be inverse to the *in situ* staining pattern. The different background colors are an artifact and not part of the model. The bordered factors are the centroids of the largest clusters, while representative occurrences of genes shared among clusters are indicated by the weighted lines.

sBFA was successful in automatically extracting interpretable patterns based on our choice of pixel intensities as input features. Figure 2 illustrates this for an example grid size of 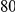
×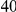 and 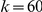 factors, and the estimated sparse factor loading matrix is shown in Figure S1. In particular, many factors correspond to prototypical lateral view patterns along the anterior/posterior axis, reflecting the activity of the segmentation network. Others represent expression differences along the dorsal/ventral axis, and patterns from different views, showcasing the ability of the method to automatically extract distinct patterns for different embryo orientations. In addition, some factors do not represent distinct expression patterns but rather the embryo shape or lighting artifacts. While these factors certainly reflect commonalities among the input data, they show the potential of sBFA to automatically separate meaningful patterns from noise.

**Figure 3:**
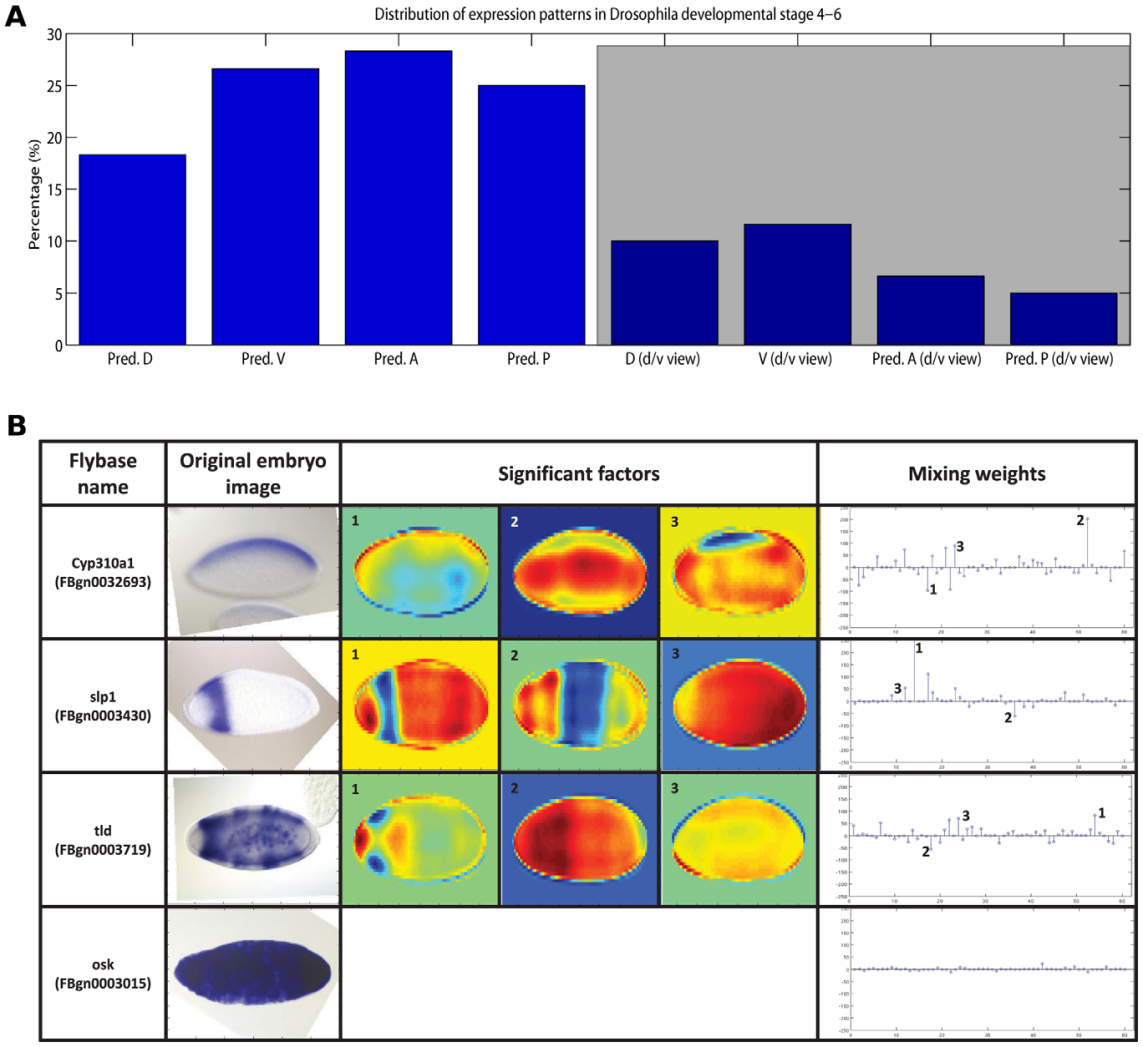
Distribution of expression patterns in Drosophila image data from stages 4–6. (A) Distribution of gene expression patterns. The height of the bars corresponds to the percentage of patterns in the indicated direction (lateral view for the first 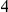 bars and dorsal/ventral view for the remaining 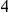 bars, shaded area). Pred. D = predominantly dorsal (lateral view), Pred. V = predominantly ventral (lateral view), Pred. A = predominantly anterior (lateral view), Pred. P = predominantly posterior (lateral view), D (d/v view) = dorsal (dorsal/ventral view), V (d/v view) = ventral (dorsal/ventral view), Pred. A (d/v view) = predominantly anterior (dorsal/ventral view), Pred. P (d/v view) = predominantly posterior (dorsal/ventral view). (B) Example factor contributions. The top three rows show significant factors contributing to the original image decomposition, for lateral (anterior/posterior) and dorsal/ventral gene expressions. The bottom row corresponds to a non-informative (maternal expression only) case, where all factors share similar low weights for their image decomposition. Overall, for any given image (arbitrary orientation), factors which show that particular gene expression orientation are more likely to contribute to the image decomposition, through more informative weights.

Besides image reconstruction, the factor loading matrix provides for an elegant way for clustering and co-expression analysis: the factors (rows in the factor matrix 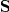) represent cluster centroids and the mixing weights (entries in the factor loading matrix 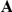) describe co-expression between genes. Each cluster can then be referred to through its corresponding factor. To illustrate this, we selected the entry/factor in the factor loading matrix with the highest absolute value for each gene in data set 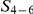. The resulting clusters divided the expression landscape into distinct categories, defining clusters of genes with various expression patterns. Compared to Frise et al. [17], who illustrated the correspondence of clusters to a developmental fate map, the sBFA framework was thus able to discover highly similar expression domains and the underlying relationships among them, but with no prior manual initialization. Within the largest clusters (Figure 2), we noticed broadly expressed genes, anteriorly expressed genes, posteriorly expressed genes, as well as dorsal/ventral expression. We further investigated co-expression by identifying instances where two clusters shared genes (two columns in the factor loading matrix contain informative mixing weights for common genes; for informative weights, we selected all loading matrix entries within 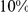 of the absolute highest value, in accordance with the sparsity assumption of the model). In most of the cases, linked clusters correspond to a general trend of temporally progressing gene expression, from larger expression domains to more narrowly defined spatial expression (Figure 2). Categorizing the factors revealed that among lateral views, a larger number of genes in the data set were expressed anteriorly and ventrally, and fewer genes posteriorly and dorsally (Figure 3A). Among dorsal/ventral views, most of the expressed genes have ventral view and predominantly anterior orientation.

As mentioned earlier, data set 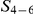 covered 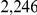 images with arbitrary orientation (lateral, dorsal/ventral). The inferred set of factors and factor loading matrix unveiled another important strength of the proposed framework: for any given image, factors which represent the same embryo orientation are more likely to contribute to the image decomposition, through more informative weights. As a result, estimated factors that show a clear lateral gene expression would be highly used by lateral gene expressed images in their corresponding factor linear combination; furthermore, estimated factors with dorsal/ventral expressions would be most likely used by dorsal/ventral input gene patterns. The four examples in Figure 3B illustrate lateral, dorsal/ventral, and non-informative expression. As expected, for non-informative maternal expression, all factors share relatively low weights in their image decomposition.

**Figure 4:**
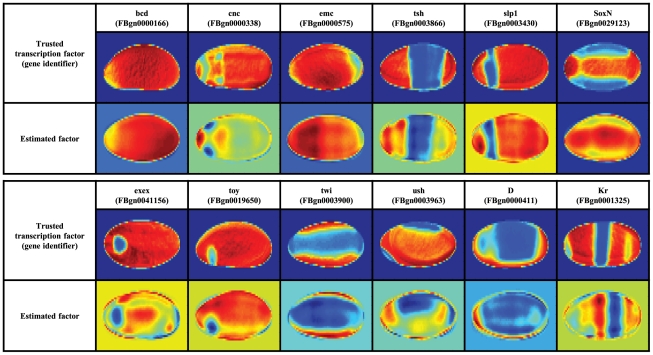
Similarities between estimated factors in 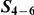 and entries in the FlyTF database.

As co-regulated genes are frequently co-regulated by transcription factors, we next inspected the similarities between estimated factors (matrix 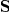 in our model) and the FlyTF database of *Drosophila* site-specific transcription factors [20]. This database contains 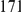 manually annotated site-specific transcription factors, identified from a list of candidate proteins with transcription-related GO annotation as well as structural DNA-binding domains assignments. Careful visual inspection revealed that a number of inferred factors were close to the expression patterns of the 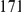 experimentally verified transcription factors (Figure 4), thus suggesting that the model factors are reflecting underlying biological functions. Moreover, the majority of the discovered similarities (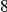 out of 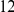 cases) correspond to the top ranked factors shown in Figure 2.

### Biological correlates of inferred factors

**Figure 5:**
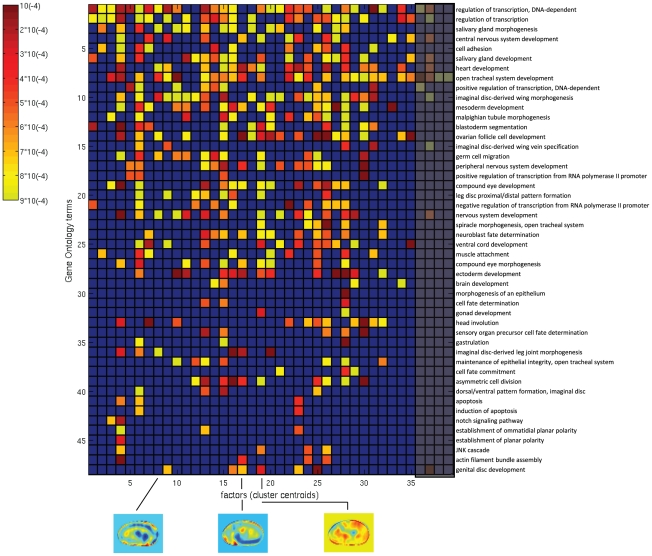
Enrichment of GO terms in the biological process (GO:0009987) category, for 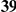 representative factors (cluster centroids), developmental stages 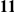
–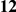. The level of significance of each GO term (vertical axis) is displayed as color intensity between yellow (p-value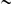 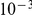) and red (p-value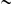 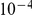), as indicated by the color bar on the left side; smaller p-values correspond to more enriched genes. The blue color corresponds to GO terms with a p-value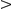 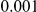.

Clusters of co-regulated genes inferred from microarray analyses have frequently been shown to reflect groups of genes with distinct functions. A popular approach is to determine enrichments of functional annotations, such as provided by the Gene Ontology, to genes within each cluster. For this aim, we selected the 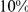 absolute highest value entries from the factor loading matrix to find enriched GO biological process terms (corrected p-value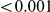 for hypergeometric test). The early development during stages 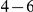 is largely centered on specifying the body axes and layout, and we thus examined the later stage data set 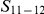 which included a broader range of ontology terms (Figure 5). Compared to the stage 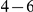 analysis, we used a larger matrix with 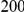 factors to allow for the identification of a larger number of distinct patterns.

Among the entire selection of biological process terms (GO:0009987), we found 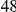 biological processes with significant enrichments mapping to one or more of 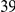 out of 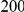 clusters. In particular, cluster 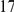 had a clear enrichment of genes with heart development function (GO:0007507) which agrees with the gene expression showed by the factor itself (at stage 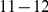, heart precursors have been specified within the dorsal mesoderm). Cluster 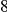, with a pattern localized around the germ band, is highly enriched in germ cell migration genes (GO:0008354). Finally, cluster 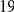 shows central/posterior development, related to the enrichment of genes with gonad development function (GO:0008406).

**Figure 6:**
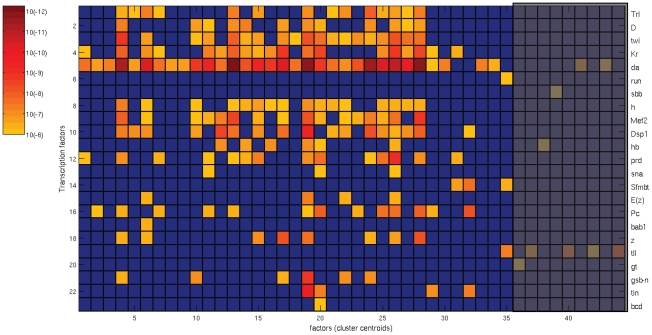
Significant transcription factors, for 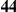 representative factors (cluster centroids), developmental stages 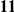
–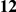. The level of significance of each TFs (vertical axis) is displayed as color intensity between yellow (p-value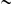 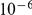) and red (p-value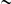 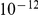), as indicated by the color bar on the left side; smaller p-values correspond to more significant genes. The blue color corresponds to TFs with a corrected p-value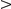 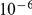.

The availability of recent genome-wide regulatory information made it possible to additionally investigate regulatory relationships between transcription factors and their target genes. Using the same clusters as for the GO enrichment analysis, we examined the agreement of factors with the “physical” regulatory network published by the modENCODE consortium [21]). This static network was inferred from 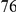 TFs with experimentally derived binding profiles, combining chromatin immunoprecipitation data from multiple cultured cell lines with chromatin information and conserved sequence elements. It covers more than 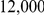 target genes; on average, genes were targeted by 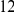 TFs, with up to 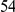 regulatory inputs. We carefully selected the subset of TFs with demonstrated expression during *Drosophila* embryogenesis as profiled in the BDGP database as well as FlyBase, and identified the significant ones for every set of genes with high value entries in the factor loading matrix (following the GO analysis described before). For developmental stages 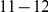, we found 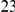 significant TFs (corrected p-value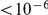 for Pearson's Chi-square test) mapping to one or more of 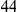 out of 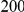 clusters (Figure 6). Out of these 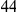 significant clusters, 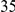 are shared with the clusters found in the GO analysis (Figure 5). There are 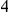 clusters that only show significant enrichments among biological functions and 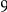 clusters with solely significant TFs (shaded areas). Nevertheless, most of the clusters of interest share biological functions as well as physical regulatory relationships and illustrate a strong consistency between the two analyses.

Moreover, clusters with significance for both biological function and transcription regulation revealed term associations between transcription factors and biological processes currently not found in the Gene Ontology database. For instance, Trl targets (FBgn0013263) are enriched in germ cell migration (cluster 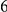) and heart development (cluster 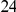); Trl mutants have been reported to exhibit defects in oogenesis [22]. Twi targets (FBgn0003900) are associated with cell adhesion (cluster 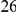), consistent with findings from genome wide ChIP analyses [23]; a complete list with term associations between transcription factors and biological processes can be found in Figure S2.

To put these results into context, we identified the set of modENCODE TFs enriched within the gene sets of the 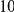 most frequent developmental terms of the controlled vocabulary as annotated by human experts (Figure S3). Among the 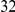 enriched TFs, a subset of 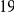 TFs are shared with the sBFA cluster-based transcription regulation analysis. The TFs that were only identified in the CV analysis are mostly general regulators; *e.g.* involved in chromatin remodeling and silencing (trx, BEAF-32, CTCF, TfllB, or CBP). These enrichments are not function-specific and therefore spurious hits. On the other hand, there are only four TFs specific to the sBFA cluster-based analysis: among them, bab1 targets (FBgn0004870) are enriched during ectoderm development, consistent with recent reports based on sequence motif analyses [24]. The automatically inferred factors are therefore more enriched in specific TF targets, and lead to a cleaner and more extensive set of links between TFs, expression patterns, and biological functions.

Lastly, we visually inspected similarities between spatial expression of estimated sparse model factors (cluster centroids) and corresponding TFs with significant p-values. Three example cases are shown in Figure S4, and they suggest that the estimated factors not only reflect biological functions but also explain correlations within the physical regulatory network.

In conclusion, our method can be used to find physical/functional networks that are relevant to *Drosophila* embryonic developmental stages of interest. In this case, the network associated to stages 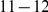 appears to be a highly modular cohesive component of the full physical regulatory network introduced in [21]; the multitude of highly significant TFs advance the hypothesis of a self-contained, highly evolvable structure.

### Gene classification into developmental expression domains

While gene expression data is often analyzed in an unsupervised fashion, the expert annotation of images with anatomical terms also allows for a direct evaluation whether extracted features reflect distinct biological patterns. To demonstrate the effectiveness of the sparse factor analysis in exploiting the hidden structure shared among different genes, entries in the factor loading matrix (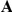) were subsequently used as features by two state-of-the-art classifiers: the SVM (polynomial kernel) [25] and a sparse multinomial logistic regression model, SMLR [26].

In evaluating the relative performance of the classifiers for individual annotation terms, we trained binary classifiers, one for each anatomical annotation term. We only considered terms associated to more than 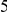 genes; terms with too few annotated genes were statistically too weak to be learned and evaluated effectively (for the developmental stages 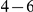, this selection translated into removing 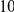 of the initial non-trivial terms mentioned before). For each of these remaining terms, the question was whether the factor loadings would be effective features to discriminate genes with a particular annotation term from those without one (to automatically identify the anatomical regions that express a gene, given a training set of annotations). We chose sparse classifiers, as some factors appeared to reflect common sources of noise (*e.g.* illumination differences) and should thus be uninformative for annotation. The accuracy of sBFA-based classifiers is represented by the area under the ROC curve (AUC values, [27]).

**Figure 7:**
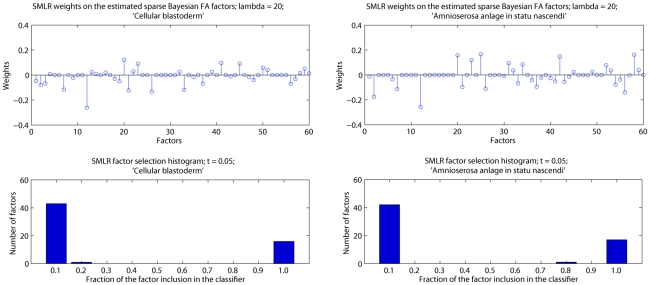
SMLR analysis on the estimated sBFA factors on data set 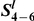, for two randomly selected annotation terms. The top row shows the SMLR mixing weights on the factors, for a regularization parameter 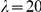; the x-axis represents the FA factors: the first 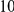 factors for a grid size of 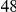
×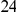, the next 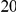 factors for a grid size of 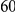
×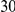 and the last 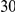 factors for a grid size of 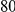
×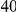. The bottom row contains histograms with the number of factors selected as relevant over 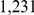 LOO-CV trials, with a cut-off value at 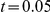. Each feature appears once in the graph. The more mass concentrated at the two ends, the more consistent the classifier is in identifying relevant factors.

We started with data set 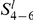, which contained 1,231 genes annotated with a total of 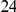 terms, and the SMLR classifier, which allows one to assess the importance of features for a classification task by the weights assigned to each feature. We first analyzed the SMLR weights 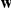 on the entire set of features (three different resolutions with corresponding number of factors of 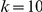, 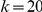 and 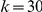 leading to a combined 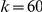 factors), and examined the number of times factors were selected as relevant by the SMLR algorithm during leave-one-out cross-validation (LOO-CV). During cross-validation, all images corresponding to a single gene were left out and the model was trained on the remaining set of images. A few common factors were not selected as relevant by any annotation term model, which confirmed our initial belief that some factors were uninformative for at least some annotations. In addition, there is strong consistency in factor selection, and most factors are either always or never included. Figure 7 shows the mixing weights on the factors for two randomly selected annotation terms, as well as a histogram of the number of times each factor is selected as relevant over the entire set of 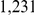 trials, with a cut-off value for feature selection at 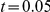. Specifically, for the ‘amnioserosa anlage in statu nascendi’ annotation term, 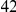 factors were never selected while 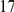 were always selected.

**Figure 8:**
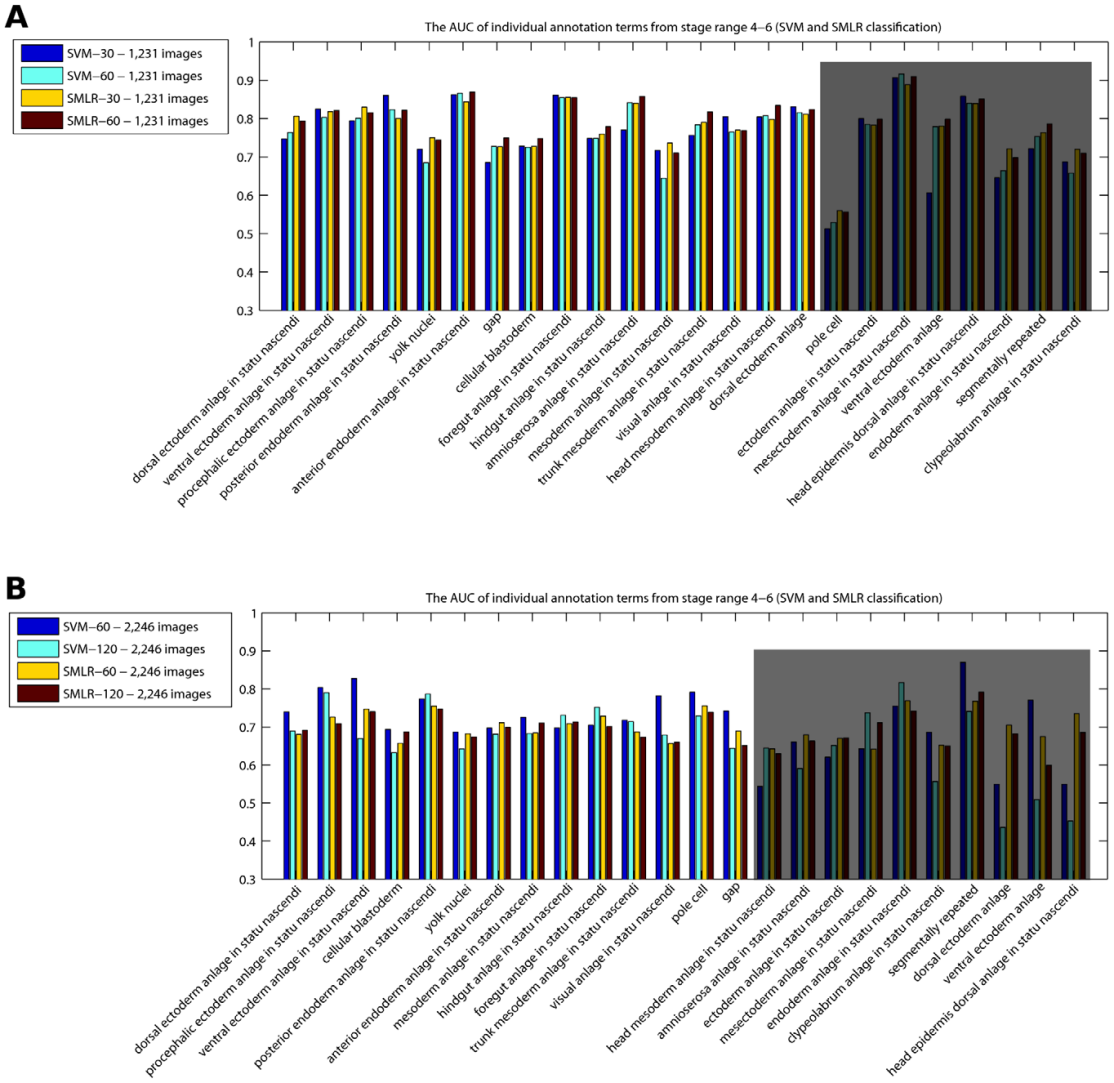
SMLR and SVM comparison on (A) data set 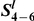 and (B) data set 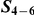: the AUC of individual annotation terms from the time window of developmental stages 4–6. (A) We consider two different scenarios: using the factors corresponding to the highest resolution, 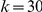 (SVM-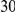 and SMLR-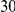), or using the entire set of factors available (SVM-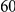 and SMLR-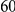). The last 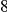 annotation terms correspond to 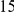 genes or less, too few to count for a strong statistical evaluation (shaded area). (B) We consider two different scenarios: using the factors corresponding to the highest resolution, 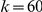 (SVM-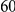 and SMLR-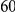), or using the entire set of factors available (SVM-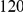 and SMLR-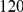). The last 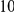 annotation terms correspond to 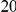 genes or less, and results are less reliable due to the stronger variance and impact of results on individual samples (shaded area).

To evaluate the success of annotation prediction, we computed AUC values achieved by the SMLR framework on data set 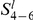 using LOO-CV (Figure 8A). To assess the influence of a particular classifier, we compared the SMLR results to those achieved by polynomial SVMs. The AUC value for each annotation term was computed using majority voting across all genes (see ‘Materials and Methods’). We see that on average, the annotation process reached similar performances with both classifiers, above 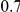 across all terms (exception are the ‘pole cell’ and ‘ventral ectoderm anlage’ annotation terms; the ‘pole cell’ lower performance can be explained by the fact that these germline precursor cells migrate and may have little overlapping spatial expression during stage 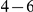).

In the next phase, we evaluated the effect of integrating images with multiple views at early stages in *Drosophila* development, by running the sBFA on data set 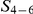; as previously mentioned, it covers 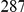 genes (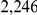 images) with arbitrary orientation (most lateral and dorsal/ventral). Similar to the previous case, we carefully examined different numbers of factors for different image resolutions and observed the following good matches: 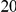, 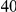 and 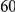 factors for a grid size of 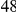
×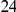, 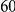
×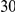 and 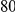
×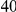, respectively. On the images in this set, the SVM-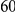 slightly outperforms the SVM-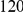 and both SMLR-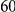 and SMLR-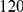 results and leads to overall consistent results despite the large variety of patterns, inconsistency among patterns associated with the same term, and variable orientation (Figure 8B). AUC values fall largely between 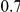 and 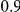 with a few exceptions, where we believe that either the annotation terms were assigned to the wrong images, or the corresponding images had some tilted viewing angle, making the understanding of the 2D pattern difficult to accomplish. Figure S5 shows two scenarios where several images corresponding to the same genes are either uninformative, out of focus or under tilted viewing angles, or show expression at different time points, making it impossible for an automated annotation process to reach perfect accuracy.

To assess how the good performance of the sBFA model would translate to later development, we applied it to the full set of images from stage 11–12 (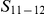), representing a more complicated image annotation problem, given the variety of orientations (lateral, dorsal/ventral) and very intricate spatial expression patterns. The sBFA framework was run on both the complete data and the lateral subset; classifiers were trained/tested on the top 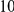 most frequent annotation terms. As the above results did not show a clear advantage of using features from multiple resolutions, we used the highest resolution (grid size) of 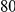
×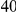 on the complete set, and a total number of factors 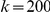. Due to the larger number of images, training and test data sets were generated 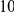 times by randomly selecting 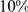 each with and without a specific annotation from the total set of 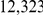 images. On the set of lateral view images only (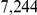 images), the sBFA model was run on the same grid-size and a smaller number of factors 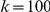; in this case, the training and test data sets were generated 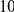 times by randomly selecting 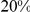 from lateral views (to achieve a comparable number of images between the two scenarios). The AUC values for each annotation term obtained by the sBFA framework (SVM classifier) were computed using both minority and majority voting, i.e. counting a gene as a true positive hit if it had at least one of its images, or the majority of images, correctly classified. According to our expectations, minority voting reaches AUC values of 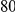
–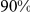, with a high 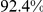 performance corresponding to ‘posterior midgut primordium’. When using majority voting, the performance is in the same range (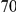
–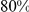) as on the images from early development, this time with a slight advantage of SMLR over SVM, indicating that sBFA was successfully able to represent more complex expression patterns (Table 1).

**Table 1:** Annotation performance in terms of AUC (mean and standard deviation), using the LOO-CV scheme, data set 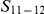.

Classifier	PMP	AMP	BP	VNCP	TMP	HPP	DEP	FP	HMP	SMP
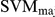	77.4 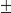 0.71	77.1 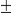 0.39	75.2 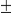 0.97	73.9 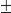 1.12	74.3 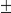 1.03	75.8 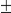 0.86	73.5 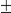 0.28	73.2 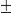 0.33	73.7 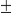 0.58	73.9 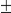 1.32
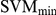	92.4 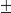 1.74	90.6 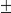 1.19	89.7 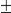 1.26	87.6 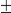 0.65	82.2 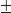 0.78	83.4 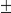 1.12	79.9 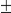 1.70	79.7 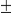 1.11	77.1 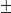 1.09	80.8 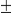 1.48
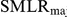	77.5 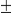 0.43	76.6 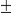 0.52	77.2 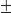 0.64	74.1 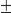 0.36	74.8 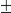 0.42	76.2 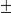 0.26	75.2 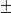 0.77	74.5 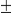 0.65	74.8 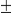 0.68	75.1 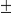 0.83
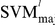	71.2 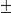 1.14	72.5 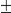 1.49	71.1 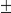 0.96	72.8 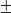 0.91	72.2 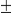 0.73	70.6 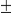 1.27	70.1 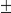 1.41	67.3 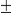 1.44	70.4 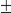 0.72	69.6 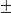 1.29
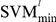	87.4 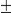 0.88	88.1 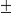 1.31	87.5 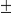 1.74	86.1 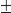 0.82	81.1 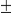 0.52	80.3 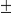 1.05	77.5 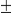 1.46	80.6 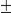 1.08	75.8 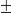 1.35	78.9 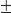 0.55
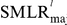	70.5 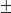 0.95	72.1 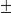 0.57	73.5 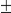 0.88	73.7 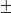 0.38	72.9 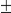 0.74	71.1 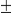 0.48	71.9 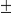 1.01	69.5 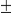 1.18	72.2 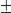 1.22	69.7 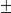 1.23

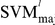
, 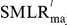 and 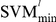 denote the performance obtained by the SVM and SMLR classifiers on lateral view images only, using both majority (maj) and minority voting (min). For more details on majority and minority voting, please see ‘Materials and methods’. For each case, 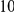 random partitions of the training and testing data sets are generated, on the 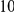 most popular annotation terms. Abbreviations of the anatomical annotations: AMP - anterior midgut primordium; BP - brain primordium; DEP - dorsal epidermis primordium; FP - foregut primordium; HMP - head mesoderm primordium; HPP - hindgut proper primordium; PMP - posterior midgut primordium; SMP - somatic muscle primordium; TMP - trunk mesoderm primordium; VNCP - ventral nerve cord primordium.

The overall improved performance of minority over majority voting (in the range of 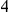
–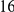 AUC percent points) is a direct reflection of the nature of the actual images used by our model. For a given gene, this can happen when most, but not all, of the images are of poor quality (out of focus, poor quality of staining/washing); the existence of at least one clear and representative image can lead to a successful minority classification. Additional complications arise from errors in the automatic normalization (such as incorrect orientation), and outlier images from different views. Several such examples are shown in Figure S6: gene FBgn0033227 is annotated with ‘posterior midgut primordium’ on a total of three images, two of which were impossible to classify due to poor quality staining and washing; FBgn0002174 is incorrectly annotated on a total of three images, two of which contain non-informative patterns; FBgn0015774 was incorrectly majority voted for two different controlled vocabulary terms, in both cases, images are either out of focus, with non-informative patterns or improperly rotated by the automated registration process.

The analysis of integrating images with multiple views revealed that, for stages 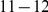, the annotation performance consistently increased when incorporating images from views other than lateral. In comparison, the average AUC performance on the lateral view only data set from stages 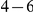 slightly outperformed the annotation using multiple views. In 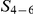, the additional views increased the number of genes as much as the number of images, meaning that most genes were represented by either lateral or other views. Additional dorsal/ventral view images are less informative for annotating purposes during early stages in *Drosophila* embryogenesis, which generally follows simple expression dynamics oriented along the A/P or D/V axis. In contrast, at later developmental stages with more complex patterns, the dorsal/ventral view images become more informative for embryo annotation, as certain expression patterns cannot be fully represented by one 2D view only.

In summary, our results confirm that a fully automatic image analysis pipeline without any human intervention can lead to highly successful expression pattern classification, despite variations in orientation and the presence of uninformative images and/or registration errors. Since both classifiers (SVM and SMLR) achieved similar annotation results, it further demonstrates the general effectiveness of the sparse Bayesian factor representation.

### Comparison to previous automatic annotation efforts

To put our approach in context, we compared our results to two state-of-the-art systems representing bottom-up approaches using many low-level features. The automatic image annotation platform IANO was introduced by Peng *et al.* [15]; in the original study, it used three different feature representations and several classifiers to predict annotations, which were reported on lateral-view images only. To provide for a fair comparison on the same set of genes, we ran the first comparison on data set 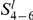, using the IANO code as provided by the authors. In its current version, SVMs were the only available classifier; furthermore, binary prediction labels were provided, which prevented the use of AUC as evaluation metric. Instead, we followed the authors' example and used the absolute recognition rate, despite its flaws on unbalanced data sets which leads to inflated results, as opposed to the balanced view obtained by AUC (for more details, see ‘Materials and Methods’). With this in mind, the results from both sBFA (majority voting) and IANO systems on the 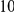 most frequent annotation terms showed that the sBFA model clearly outperformed IANO (Table 2), at lower dimensionality. The proposed sBFA model consists of a fixed grid-based feature extraction technique followed by a sparse Bayesian factor analysis framework, whereas IANO considers three local and global feature extraction analyses which might result in higher-dimensional feature spaces.

**Table 2:** Overall recognition rate (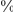) of the sBFA and IANO models, data set 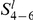 (stages 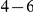).

Classifier	DEASN	PrEASN	VEASN	CB	PoEASN	YN	AEASN	MASN	HASN	FASN	Mean
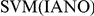	53.8	56.5	58.2	77.1	77.2	78.6	76.8	77.3	78.7	80.4	71.46
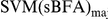	65.4	67.9	68.1	80.2	81.7	78.5	81.2	84.4	86.3	85.9	77.96

Image level recognition rates on the top 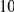 most frequent annotation terms from the time window of developmental stages 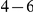; majority voting (maj) was used for the sBFA model. Abbreviations of the anatomical annotations: AEASN - anterior endoderm anlage in statu nascendi; CB - cellular blastoderm; DEASN - dorsal ectoderm anlage in statu nascendi; FASN - foregut anlage in statu nascendi; HASN - hindgut anlage in statu nascendi; MASN - mesoderm anlage in statu nascendi; PoEASN - posterior endoderm anlage in statu nascendi; PrEASN - procephalic ectoderm anlage in statu nascendi; VEASN - ventral ectoderm anlage in statu nascendi; YN - yolk nuclei.

The original IANO results focused on a manually selected data set of 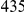 representative gene images with lateral views from stages 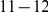 [15]. While we were able to obtain identifiers for the genes, the exact images used in their work were no longer available from the authors; as a result, for the second comparison, we considered all BDGP images from stage 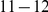 for the 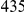 genes (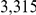 images, data set 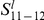). Using sBFA with a polynomial kernel SVM classifier, we obtained results using both minority and majority voting. The average recognition rate for the 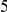 annotation terms evaluated by Peng *et al.* are shown in Table 3; minority voting 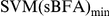 is the measure most likely to recapitulate the IANO results reported for the smaller, manually curated data set [15]. Altogether, sBFA lead to clearly improved results when applied on the same data sets, or on a prediction scheme aimed at recapitulating the original scenario, demonstrating the robustness of our generative feature extraction method when using SVM classifiers.

**Table 3:** Overall recognition rate (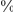) of the sBFA and IANO models, data set 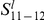 (stages 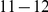).

Classifier	HPP	PMP	AMP	PP/BP	DEP	Mean
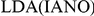	83	80	84	86	88	84.2
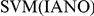	91	89	91	97	95	92.6
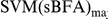	90.1	85.5	86.4	78.3	93.9	85.8
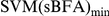	96.5	95.3	95.1	95.8	98.1	96.16

The updated controlled vocabulary replaced the PP (protocerebrum primordium) annotation term with BP (brain primordium); minority voting 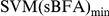 is the closest measure to the SVM(IANO) results based on a manually selected data set. In the one case where our performance ranks below IANO, numbers may not be exactly comparable, as the updated database release we used had rephrased the ontology term and reannotated some images. Abbreviations of the anatomical annotations: AMP - anterior midgut primordium; BP - brain primordium; DEP - dorsal epidermis primordium; HPP - hindgut proper primordium; PMP - posterior midgut primordium; PP - protocerebrum primordium.

A more recent study used dense Scale-Invariant Feature Transform (SIFT) descriptors [28] that were converted into sparse codes to form a codebook to represent registered images, and proposed a local regularization procedure for the learning process [14]. An unbiased comparison between our model and this system was hard to establish since the image IDs were not published in detail, results were based at least partially on selected orientations and not full sets, and annotation terms did not exactly correspond to the BDGP ontology. However, our results based on a much smaller feature space (effectively around 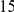 features for the SMLR classifiers, as opposed to several thousand), are in a similar range to the ones reported by their system.

## Discussion

Digital images are a quickly increasing new source of data for problems in computational biology. Given the very diverse nature of imaging technology, samples, and biological questions, approaches are oftentimes very tailored and ad hoc to a specific data set. At the same time, high content screening of phenotypes is moving from cell-based assays to whole organisms, and phenotypes can no longer be manually annotated due to large volumes of data. In this paper we presented a general method for the automatic decomposition of spatial quantitative information, applied on the dissection and annotation of gene expression images. The algorithm is based on a fully Bayesian factor analysis formulation, and annotates images based on a trained SVM or SMLR model. We also employed the biologically justified prior assumption that the models for both factor inference and classification are sparse, implying that only a small subset of factors are used to define expression domains. Indeed, the classifiers make use of only a dozen or two of features, orders of magnitude less than state-of-the-art approaches addressing the same problem. We also demonstrated that genes with strong weights to the same factor share specific biological functions or are targets of the same transcription factor, providing important starting point for future in-depth analysis.

Our approach is probably closest to Pan *et al.* [29], which introduced an image mining system to discover latent spatial “themes” of gene expressions, by using PCA and independent component analysis (ICA) based features. ICA assumes independence at the regulatory level, and the resulting decomposition may lack the physical or biological association to sBFA factors, by not imposing sparsity within the model (as the biological prior assumption). Unlike PCA, sBFA includes sparseness constraints and allows for independent additive measurement errors on the observed variables. Whereas the earlier study was mostly exploratory and did not include a specific application, we provided extensive results on fruit fly embryonic expression pattern annotation from early and late stages.

Our results showed that sBFA automatically identifies and separates patterns corresponding to different views, and subsequently makes successful predictions even when presented with images of the same gene taken from different angles. In addition to the automatic pattern separation, factor loadings can also automatically identify and filter non-informative (such as ubiquitous) gene expression patterns. To illustrate this, we manually selected a set of 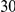 informative images (lateral, dorsal/ventral expression) and 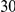 non-informative images (mostly maternal expression) from data set 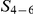 and computed the Euclidean distances between their corresponding estimated sparse mixing weights (rows in matrix 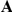) and the null vector as reference. Choosing a threshold to separate the informative images from non-informative images (please see Figure S7), we succesfully filtered the original data set 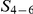 by removing a total of 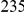 non-informative images (about 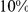 of the total number of images). The subsequently obtained AUC values on the filtered data set of 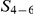 (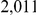 images) displayed the further improvement achieved by this simple Euclidean-based analysis (Figure S8). For future work, we plan to extend the sparse Bayesian analysis to the entire BDGP database (multiple developmental stage analysis); after extracting stage-window specific factors, a classifier taking factor weights from different stage windows as input may be able to increase the baseline performance obtained on one stage window only.

In constructing the sparse FA representation, we only used simple grid-based features. This provided for an easy human interpretation of the factors and was possible because we used previously registered data as input. However, results from other groups have shown that multiple feature integration (over features including wavelet and rotation/translation invariant coefficients) can improve performance. Given that we are addressing spatial patterns, features can also take the correlation to neighboring features into account (cf. Frise *et al.*, [17]). As preliminary example, we ran the sparse Bayesian factor analysis on the features used by the 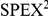 platform which was concurrently developed to our system [30]. 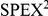 registers every raw *Drosophila* ISH image via foreground object extraction, alignment, orientation detection and concise gene expression pattern extraction (using a Markov random field model). Resulting images are then converted into a low-dimensional feature representation using the ‘triangulated image’ idea, first introduced in [17], in which embryo expression is represented on a deformable mesh of 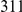 equilateral triangles in the shape of an ellipse. A comparison between the sparse Bayesian factor analysis applied on fixed grid-based filtered registered images used here [19], and the ‘triangulated’ 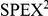 features [30], is shown in Figure S9 and Table S1. Overall, the 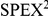 features showed a slight advantage over the fixed grid-based technique. However, the mean absolute recognition rates achieved by both feature sets when used in a sparse Bayesian factor analysis model were in a highly similar range, and filtering of non-informative images as discussed above showed a comparatively stronger positive effect. This demonstrated the robustness of the sBFA framework, and its ability to identify and separate gene expression patterns, regardless of the complexity of the feature space. In the future, application of the sBFA model on the actual set of registered 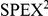 images may potentially generate accuracies even better than the mesh-based features.

The correspondence of inferred factors to expression patterns of regulatory proteins, as well as the enrichment of targets for specific TFs, suggests the potential for a more sophisticated model that incorporates known transcription factor expression patterns into the factor analysis, possibly in the shape of prior information, or as higher level information in hierarchical models. Finally, the approach can be extended by the integration of image data with other genomic sources. A previous study automatically inferred positive (spatial gene co-expression) and negative pairwise constraints (distinctive spatial expression patterns) from image data, and used them in a semi-supervised analysis of microarray time-course data [31].

In summary, factor analysis provides a flexible unsupervised framework to identify the basic vocabulary in complex image (expression) data, which leads to competitive prediction results while using only a small set of features. This sparse approach is general and applicable in other microscopy application domains, such as protein localization in subcellular domains and expression data from other organisms, and as such holds promise as a general framework for high-throughput screeening, to identify candidate gene sets with consistent altered expression under changed environmental or genetic conditions.

## Materials and Methods

### Factor analysis - model specification

Factor analysis is a statistical method first introduced by Gorsuch [32] when modeling many dependent random variables with linear combinations of a few hidden variables. Hinton *et al.* [33] pioneered an Expectation-Maximization (EM) algorithm for factor analysis in order to model the manifolds of digitized images of handwritten digits. West [34] was the first to introduce a framework for using factor analysis on gene expression data.

In matrix notation, the Bayesian factor analysis on image data can be represented as
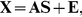
(1)where 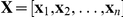 is a 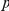 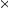 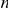 dimensional data matrix, with 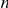 the number of features, quantifying the associated gene-expression values for 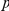 images (genes) under investigation. Each row of 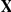 is called a *gene pattern* with dimension 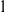 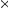 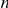. Here, we assume that each gene pattern is already normalized to zero mean. 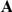 is the factor loading matrix with dimension 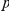 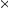 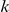, which contains the linear weights. 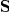 is the factor matrix with dimension 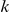 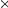 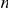, with each element modeled by a standard normal distribution. Each *column* of 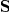 is the factor score for feature 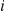 and each *row* is called a factor. 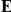 is the additive Gaussian noise with dimension 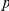 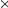 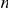. Both 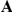 and 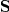 are inferred by the model simultaneously.

From the model we can see that each row of 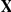 is modeled by a linear combination of the factors (rows of 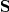), indicating that the variability of the original 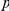 feature patterns can be explained by only 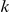 latent factors. The model can also be written in a vector form as follows
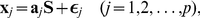
(2)where 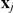 and 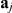 denote the 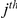 row of 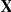 and 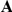, respectively and the basis matrix 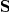 is shared across all samples. Indeed, factor analysis is an unsupervised dimensionality reduction method used widely in data analysis and signal processing [35].

To promote sparseness required by the underlying biological assumption of gene expression data, West [34] suggested the use of a mixture prior on the factor loading matrix. Thus each row of the matrix should only have a small number of non-zero elements in order to comply with the biological assumption and to make the model more interpretable. In order to follow the biological assumption where spatial gene expression patterns are modeled only by a few domains (factors), coupled with the benefit of a relatively simple inference, we employed the Student-t distribution as sparseness prior, which takes the following hierarchical form
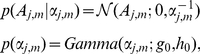
(3)with 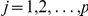, 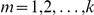, 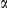 indicating the precision parameters and 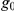, 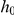 the shape and scale parameters of the gamma prior distribution on 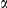. By integrating out the precision parameter 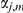, the marginal prior on 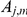 is a sparseness inducing Student-t distribution. The sparseness is controlled by the precision parameter 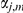. The objective of imposing this sparse prior is to automatically shrink most elements in 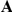 to near zero, in order to yield a more interpretable model. A comprehensive review of sparse factor analysis for gene expression data analysis is given by Pournara and Wernisch [18], with various sparse priors taken into consideration.

The full likelihood for the Student-t sparse factor analysis model can be expressed as
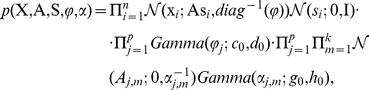
(4)where 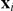 denotes the 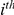 column of 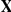, 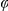 represents the precision parameters on the additive noise 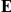, while 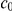 and 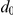 indicate the shape and scale hyperparameters on precision 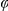, respectively.

The posterior distribution for the sparse factor analysis framework is approximated using a Markov Chain Monte Carlo (MCMC) inference.

### Detailed update equations

–Sample the factor matrix 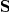 from
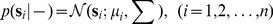
(5)where
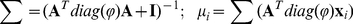
(6)and 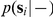 denotes conditional density of 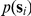 on all other random variables.

–Sample the factor loading matrix 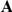 from
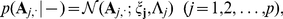
(7)where
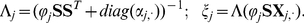
(8)Here 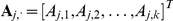 denotes the 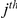 row of 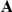; 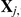 and 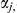 are similarly defined.

–Sample the precision parameters 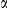 from
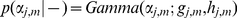
(9)with 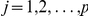 and 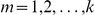, where
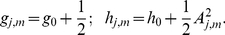
(10)–Sample the precision parameters 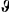 from
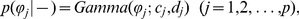
(11)where
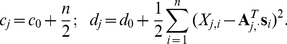
(12)


The sBFA algorithm was generally run for a total of 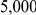 Gibbs iterations, discarding the first 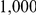 and estimating the model parameters on the remaining 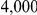 iterations. The sparse prior on the factor loading matrix (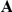) was controlled by the hyperparameter on the precision parameters 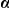, 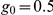, while the scale parameter of the Gamma prior distribution on 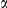 was set to 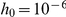. Running on a typical modern PC (Quad-core Intel Xeon 1.86 GHz processors and 4.0 GB memory), the computation times for data sets 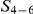 and 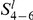 are summarized in Table 4.

**Table 4:** Representative relative CPU times of the sBFA algorithm (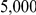 Gibbs iterations), data sets 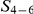 and 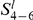.

	CPU Time (hours)	
	data set 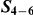	data set 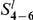
Factor Analysis (20 factors, 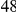 × 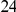 grid size)	6.1	3.3
Factor Analysis (40 factors, 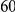 × 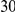 grid size)	6.9	3.9
Factor Analysis (60 factors, 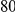 × 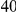 grid size)	10.5	5.1

### Automatic annotation by different classifiers

#### SVM classification

the SVM model is a supervised learning method; given a set of training examples, each marked as belonging to one of the two classes (categories), the SVM training algorithm builds a model that predicts whether a new example falls into one category or the other. Intuitively, an SVM model is a representation of the examples as points in space, mapped so that the examples of the separate categories are divided by a clear gap (decision boundary or functional margin) that is as wide as possible. New examples are then mapped into that same space and predicted to belong to a category based on which side of the gap they fall on. We used the 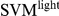 implementation [36] with a polynomial kernel function and a unity trade-off between the training error and the functional margin.

#### SMLR classification

the SMLR model learns a classifier and simultaneously performs feature selection to identify a small subset of features (factors) relevant to the class distinctions. The learned classifier reports the probability of a sample (gene expression) belonging to each of the classes given a set of feature weights, one for each class, and also estimates the mixing weights on all features. In order to achieve sparsity, the model incorporates a sparsity-promoting 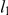 prior on the mixing weights entries and then estimates the weights using a MAP (Maximum a Posteriori) criterion. Explicitly, the model computes
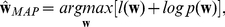
(13)where 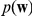 is a prior distribution on the mixing weight entries 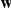 and 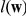 is the log-likelihood
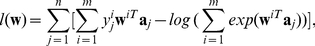
(14)with 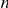 the number of training samples, 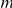 the number of classes (in our work we have a binary model, 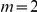), 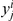 the class label for training sample 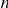 and 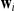 the weight vector corresponding to class 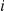.

Sparsity-promoting Laplacian priors on the mixing weights can be written as
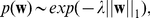
(15)where 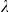 acts as a tunable regularization parameter (the larger is 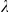, the greater is the sparsity). Excessively large values of 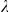 can result in the nonselection of relevant factors, while excessively small values can result in the selection of irrelevant features. We evaluated values of 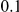, 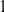, 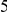, 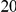 and 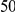 for 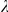 and noticed very similar best classification results at 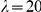 and 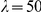, choosing 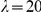 throughout the paper.

### Performance assessment

The sBFA model is demonstrated on a large set of image expression data collected within the Berkeley *Drosophila* Genome Project. The use of brightfield microscopy and the color of the staining made it hard to separate object and expression pattern from the same image, although heuristic normalization steps have been proposed [17]. We here used previously segmented and registered images [19], in which a state-of-the-art framework provided simultaneous, fully automated image segmentation and registration without human intervention. Due to the complex nature of this task, the final registration process was not perfectly accurate in terms of precise embryo extraction as well as orientation, which increased the challenge of automatic annotation. We scaled the registered images to 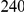
×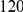 pixel resolution, containing a single embryo and no background. We defined a grid of fixed size (*e.g.*, 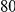
×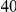 patches) and calculated the mean pixel value within each patch; all mean values were stacked into a single feature vector.

Each scaled and registered image was classified individually, with each gene being represented by one or more images. As a results of a one gene to a multiple image mapping, many earlier approaches chose representative images, one per gene, with a clearly defined informative pattern. However, for a fully automated approach, the data offer the possibility to combine results from several images to annotate a gene. The challenge is illustrated by the examples in Figure S5, which demonstrate inconsistencies and presence of noise in large image data sets; as a result, some images are more informative than others and may even lead to contradicting images within the same gene. Regardless of evaluation metric, we here use two strategies: (i) majority voting, in which a label is assigned based on the predominant label over all images associated to a particular gene; (ii) minority voting, in which a gene is considered correctly classified if at least one image has been predicted with the correct annotation term. While the latter is not a realistic metric for unseen data, it provides for a reasonably fair evaluation when comparing against previous approaches which evaluated manually selected single representative images for each gene.

The agreement between the predicted annotations and the ground truth provided by human curators was measured using AUC values for a leave-one-out cross-validation procedure. To allow for a fair comparison to previously published work, we also used the absolute recognition rate (ARR) to measure the classification accuracy. When a data set is unbalanced, this metric is not representative of the true performance of the classifier: if the larger class comprises 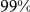 of the data, an ARR of 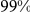 is trivially achieved by classifying all samples into the larger class. Image data sets are heavily unbalanced, as only comparatively few out of a total set of images are annotated with any given term.

## Supporting Information

Figure S1
**BDGP analysis, data set** 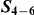 **: estimated factor loading matrix, for a grid size of 80×40, with** 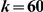 **factors.** The sBFA algorithm was run for a total of 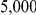 Gibbs iterations, with a burn-in of 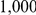 iterations. Most factor loadings are near zero (light green color), illustrating the sparseness of the solution.(TIF)

Figure S2 **A complete list with term associations between transcription factors and biological processes, developmental stages 11–12.**
(TIF)

Figure S3
**Significant transcription factors, for the top** 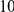 **most frequent annotation terms (developmental stages** 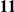 **–** 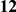 **).** The level of significance of each TFs (vertical axis) is displayed as color intensity between green (p-value 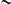 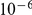) and red (p-value 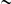 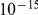), as indicated by the color bar on the left side; smaller p-values correspond to more significant genes. The blue color corresponds to TFs with a corrected p-value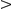 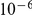.(TIF)

Figure S4
**Visual similarities between spatial expressions of estimated sparse model factors and corresponding TFs with significant p-values (data set** 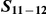 **).**
(TIF)

Figure S5 **Complexity of the image data (focal distance and viewing angles).** Two examples where several images corresponding to two individual genes (at stages 4–6 and 11–12, respectively) are either out of focus, with no visible gene expression pattern, or under tilted viewing angles, making the annotation process more difficult.
(TIF)

Figure S6
**Spatial expression patterns of genes with successful classification within the minority voting scenario, data set** 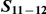 **.** Several examples where, for a given gene, there is only one correct annotated image, due to out of focus, non-informative patterns or an improper rotation by the registration process. In each case, the original gene spatial expression patterns and the automatically extracted individual embryos are shown.(TIF)

Figure S7
**Euclidean distance based informative/non-informative gene selection.** Informative images (lateral, dorsal/ventral expressions) are separated from non-informative images (mostly maternal expressions) through the use of Euclidean distance between their corresponding estimated mixing weights (rows in matrix 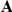) and a reference vector. The chosen threshold is further employed to succesfully remove a total of 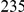 non-informative images from data set 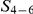.(TIF)

Figure S8
**SVM analysis on developmental stages 4–6.** The AUC values achieved by the SVM framework on the filtered data set 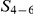 (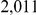 images) in comparison to the AUC results on the original data set 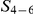.(TIF)

Figure S9
**SVM analysis on developmental stages 4–6: the AUC of individual annotation terms using the sBFA model.** We consider two different scenarios: using the grid-based features (grid size of 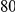
×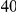), or using the 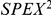-based features. The common set between the two studies extends to a total of 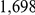 images, with different views (lateral, dorsal/ventral). During the sBFA estimation, we used a number of factors 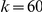, for both scenarios; the polynomial SVM generated the AUC of individual annotation terms.(TIF)

Table S1
**Overall recognition rate (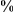) of the sBFA model on grid-based features and 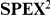-based features, developmental stages 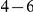.** Image level recognition rates on the top 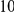 most frequent annotation terms from the time window of developmental stage 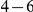 (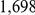 images), as used as metric in [30]. Abbreviations of the anatomical annotations: AEASN - anterior endoderm anlage in statu nascendi; CB - cellular blastoderm; DEASN - dorsal ectoderm anlage in statu nascendi; HASN - hindgut anlage in statu nascendi; MASN - mesoderm anlage in statu nascendi; PoEASN - posterior endoderm anlage in statu nascendi; PrEASN - procephalic ectoderm anlage in statu nascendi; TMASN - trunk mesoderm anlage in statu nascendi; VEASN - ventral ectoderm anlage in statu nascendi; YN - yolk nuclei.(TIF)

## References

[1] Arbeitman MN, Furlong EEM, Imam F, Johnson E, Null BH (2002). Gene expression during the life cycle of Drosophila melanogaster.. Science.

[2] Hooper SD, Boue S, Krause R, Jensen LJ, Mason CE (2007). Identification of tightly regulated groups of genes during Drosophila melanogaster embryogenesis.. Mol Sys Biol.

[3] Stolc V, Gauhar Z, Mason C, Halasz G, vanBatenburg MF (2004). A gene expression map for the euchromatic genome of Drosophila melanogaster.. Science.

[4] Tomancak P, Berman BP, Beaton A, Weiszmann R, Kwan E (2007). Global analysis of patterns of gene expression during Drosophila embryogenesis.. Genome Biol.

[5] Ljosa V, Carpenter AE (2009). Introduction to the quantitative analysis of two-dimensional fluorescence microscopy images for cell-based screening.. PLoS Comp Biol.

[6] Walter T, Shattuck DW, Baldock R, Bastin ME, Carpenter AE (2010). Visualization of image data from cells to organisms.. Nat Methods.

[7] Tautz D, Pfeifle C (1989). A non-radioactive in situ hybridization method for the localization of specific RNAs in Drosophila embryos reveals translational control of the segmentation gene hunchback.. Chromosoma.

[8] Tomancak P, Beaton A, Weiszmann R, Kwan E, Hartenstein V (2002). Systematic determination of patterns of gene expression during Drosophila embryogenesis.. Genome Biol.

[9] Carson JP, Ju T, Lu HC, Thaller C, Xu M (2005). A digital atlas to characterize the mouse brain transcriptome.. PLoS Comp Biol.

[10] Lein ES, Hawrylycz MJ, Ao N, Ayres M, Bensnger A (2007). Genome-wide atlas of gene expression in the adult mouse brain.. Nature.

[11] Ashburner M, Ball CA, Blake JA, Botstein D, Butler H (2000). Gene ontology: tool for the unification of biology - The Gene Ontology Consortium.. Nat Genet.

[12] Chen SC, Zhao T, Gordon GJ, Murphy RF (2007). Automated image analysis of protein localization in budding yeast.. Intel Sys for Mol Biol/European Conf on Comp Biol.

[13] Kumar S, Jayaraman K, Panchanathan S, Gurunathan R, Marti-Subirana A (2002). BEST: a novel computational approach for comparing gene expression patterns from early stages of Drosophila melanogaster development.. Genetics.

[14] Ji S, Li YX, Zhou ZH, Kumar S, Ye J (2009). A bag-of-words approach for Drosophila gene expression pattern annotation.. BMC Bioinformatics.

[15] Peng H, Long F, Zhou J, Leung G, Eisen MB (2007). Automatic image analysis for gene expression patterns of fly embryos.. BMC Cell Biol.

[16] Heffel A, Stadler PF, Prohaska SJ, Kauer G, Kuska JP (2008). Process flow for classification and clustering of fruit fly gene expression patterns.. IEEE Int Conf on Im Proc.

[17] Frise E, Hammonds AS, Celniker SE (2010). Systematic image-driven analysis of the spatial Drosophila embryonic expression landscape.. Mol Sys Biol.

[18] Pournara I, Wernisch L (2007). Factor analysis for gene regulatory networks and transcription factor activity profiles.. BMC Bioinformatics.

[19] Mace DL, Varnado N, Zhang W, Frise E, Ohler U (2010). Extraction and comparison of gene expression patterns from 2D RNA in situ hybridization images.. Bioinformatics.

[20] Adryan B, Teichmann SA (2006). FlyTF: a systematic review of site-specific transcription factors in the fruit fly Drosophila melanogaster.. BMC Bioinformatics.

[21] Roy S, Ernst J, Kharchenko PV, Kheradpour P, Negre N (2010). Identification of functional elements and regulatory circuits by Drosophila modENCODE.. Science.

[22] Ogienko AA, Karagodin DA, Pavlova NV, Fedorova SA, Voloshina MV (2008). Molecular and genetic description of a new hypomorphic mutation of Trithorax -like gene and analysis of its effect on Drosophila melanogaster oogenesis.. Russian J Dev Biol.

[23] Sandmann T, Girardot C, Brehme M, Tongprasit W, Stolc V (2007). A core transcriptional network for early mesoderm development in Drosophila melanogaster.. Genes Dev.

[24] Jaebum K, Cunningham R, James B, Wyder S, Gibson JD (2010). Functional characterization of transcription factor motifs using cross-species comparison across large evolutionary distances.. PLoS Comp Biol.

[25] Burges CJC (1998). A tutorial on support vector machines for pattern recognition.. Data Min Knowl Discov.

[26] Krishnapuram B, Carin L, Figueiredo MAT, Hartemink AJ (2005). Learning sparse Bayesian classifiers: multi-class formulation, fast algorithms and generalization bounds.. IEEE Trans Pattern Anal Mach Intell.

[27] Gribskov M, Robinson NL (1996). Use of receiver operating characteristic (ROC) analysis to evaluate sequence matching.. Comp Chem.

[28] Mikolajczyk K, Schmid C (2005). A performance evaluation of local descriptors.. IEEE Trans Pattern Anal Mach Intell.

[29] Pan JY, Balan AGR, Xing EP, Traina AJM, Faloutsos C (2006). Automatic mining of fruit fly embryo images.. Proc of the 12th ACM SIGKDD.

[30] Puniyani K, Faloutsos C, Xing EP (2010). SPEX^2^: automated concise extraction of spatial gene expression patterns from fly embryo ISH images.. Bioinformatics.

[31] Costa IG, Krause R, Opitz L, Schliep A (2007). Semi-supervised learning for the identification of syn-expressed genes from fused microarray and in situ image data.. BMC Bioinformatics.

[32] Gorsuch RL (1983). Factor analysis.

[33] Hinton GE, Dayan P, Revow M (1997). Modeling the manifolds of images of handwritten digits.. IEEE Trans on Neural Networks.

[34] West M (2003). Bayesian factor regression models in the “large p, small n” paradigm.. Bayesian Stat.

[35] Prince SJD, Warrell J, Elder JH, Felisberti FM (2008). Tied factor analysis for face recognition across large pose differences.. IEEE Trans Pattern Anal Mach Intell.

[36] Joachims T (1999). Making large-Scale SVM Learning Practical - Advances in Kernel Methods -Support Vector Learning.

