# Supplementary figures and images for: Automatic Annotation of Spatial Expression Patterns via Sparse Bayesian Factor Models
Source: PLoS Comput Biol. 2011 Jul 21;7(7):e1002098. doi: 10.1371/journal.pcbi.1002098 (PMC3140966; doi:10.1371/journal.pcbi.1002098)

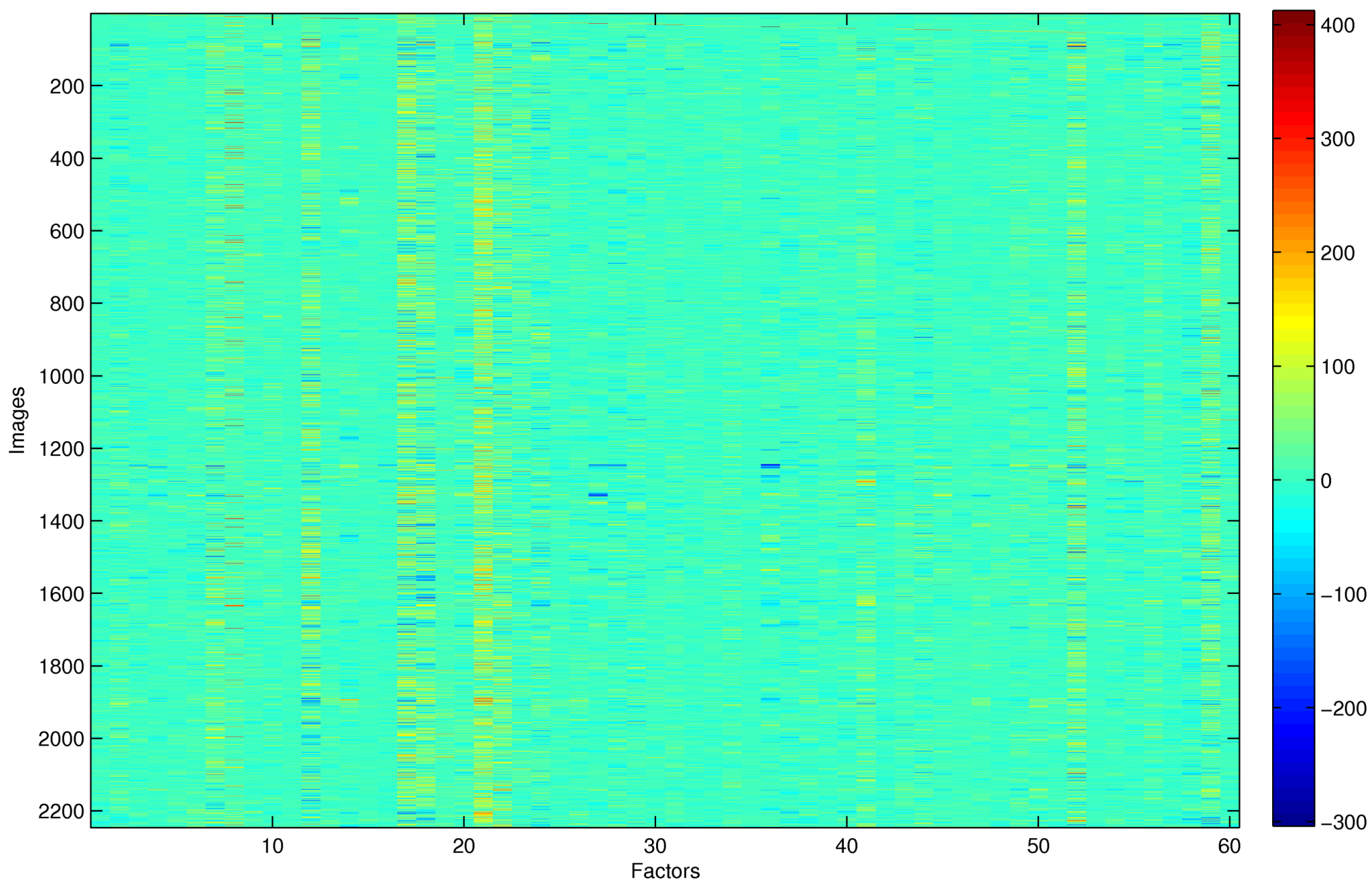

Supplement: Figure S1 — BDGP analysis, data set : estimated factor loading matrix, for a grid size of 80×40, with factors. The sBFA algorithm was run for a total of Gibbs iterations, with a burn-in of iterations. Most factor loadings are near zero (light green color), illustrating the sparseness of the solution. (TIF) [file pcbi.1002098.s001.tif]

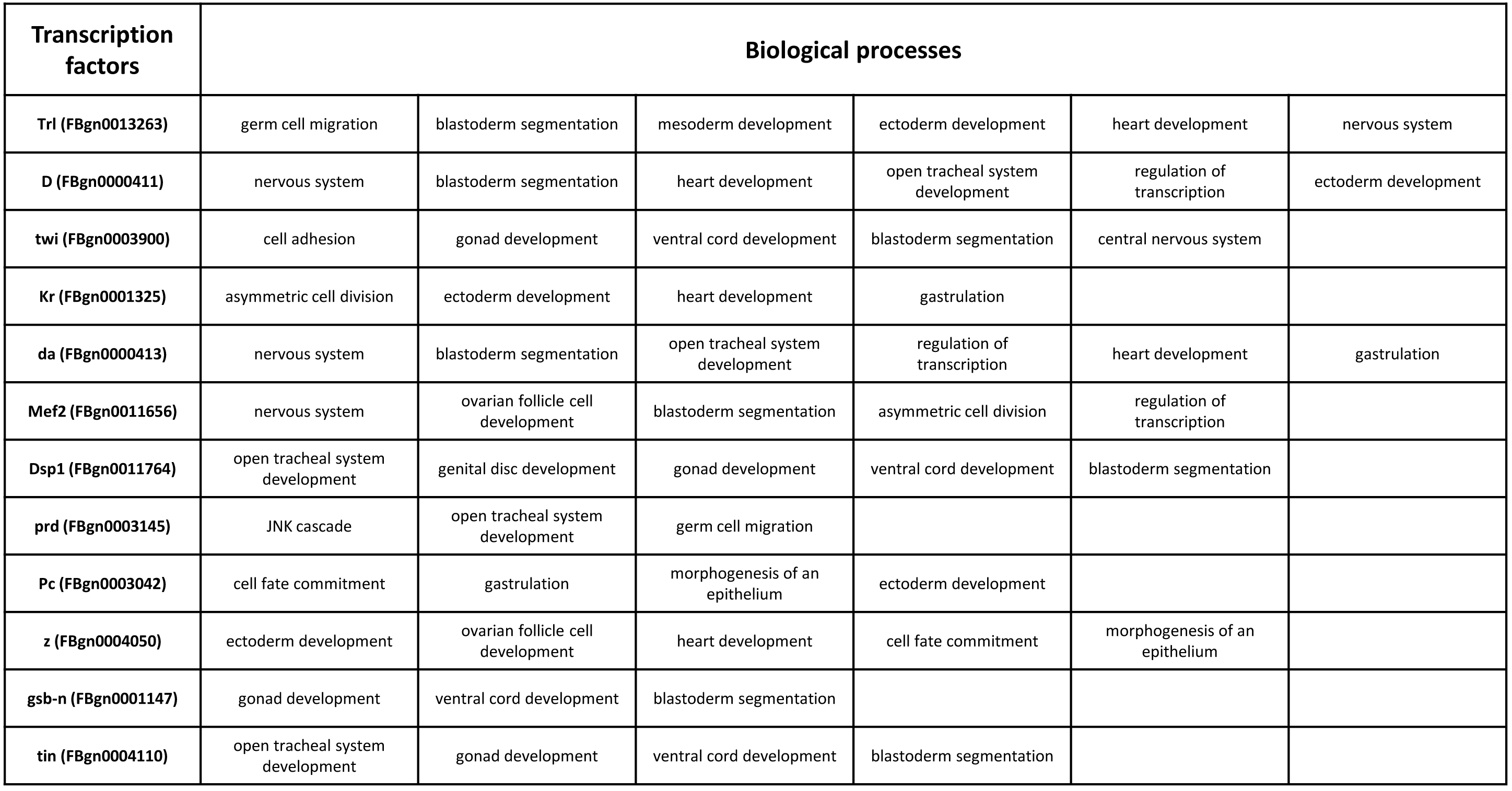

Supplement: Figure S2 — A complete list with term associations between transcription factors and biological processes, developmental stages 11–12. (TIF) [file pcbi.1002098.s002.tif]

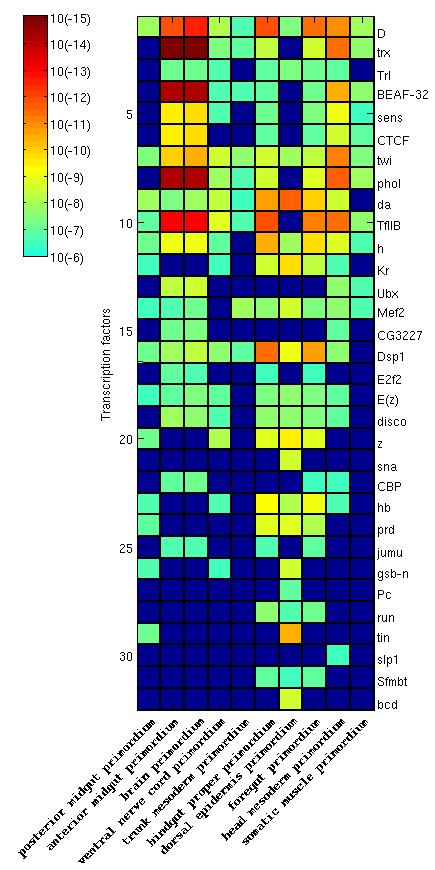

Supplement: Figure S3 — Significant transcription factors, for the top most frequent annotation terms (developmental stages – ). The level of significance of each TFs (vertical axis) is displayed as color intensity between green (p-value ) and red (p-value ), as indicated by the color bar on the left side; smaller p-values correspond to more significant genes. The blue color corresponds to TFs with a corrected p-value . (TIF) [file pcbi.1002098.s003.tif]

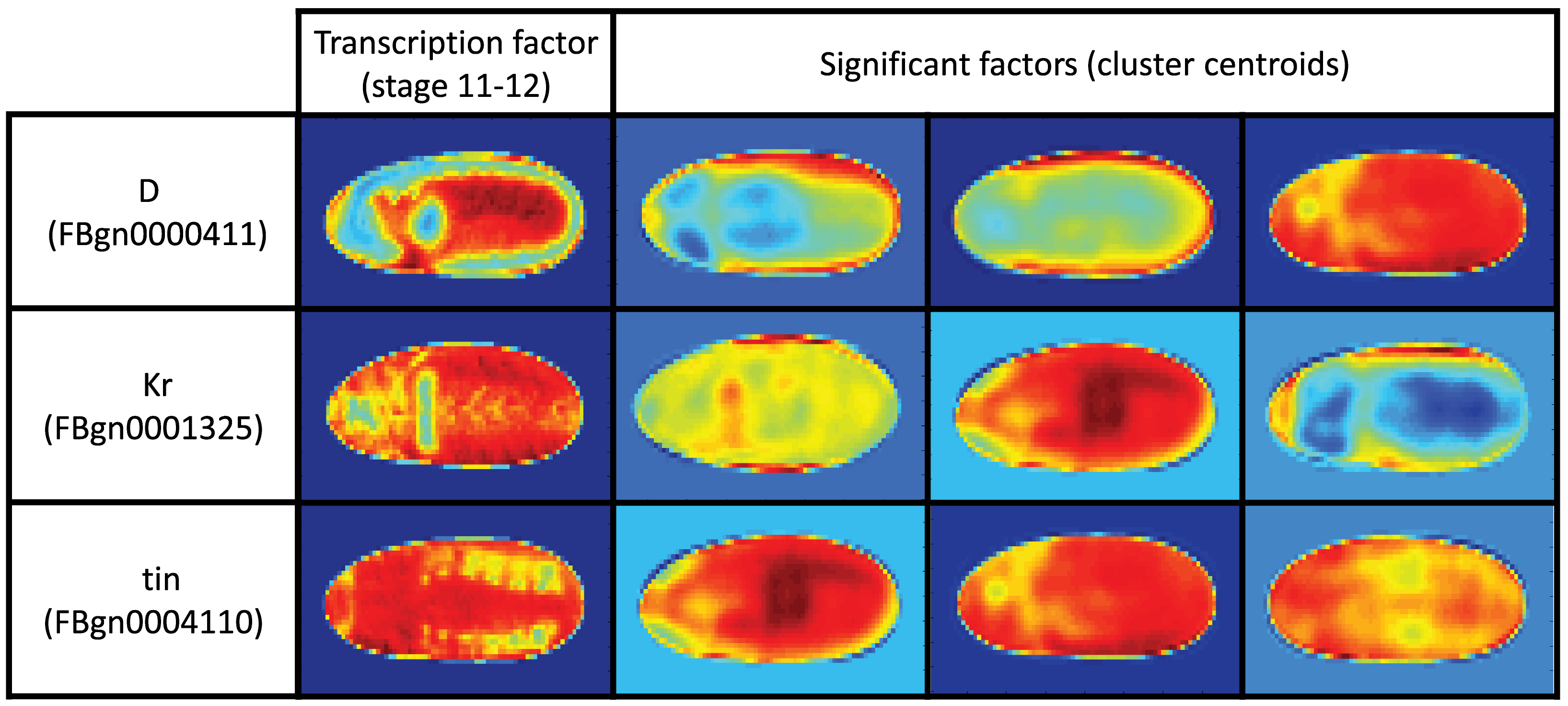

Supplement: Figure S4 — Visual similarities between spatial expressions of estimated sparse model factors and corresponding TFs with significant p-values (data set ). (TIF) [file pcbi.1002098.s004.tif]

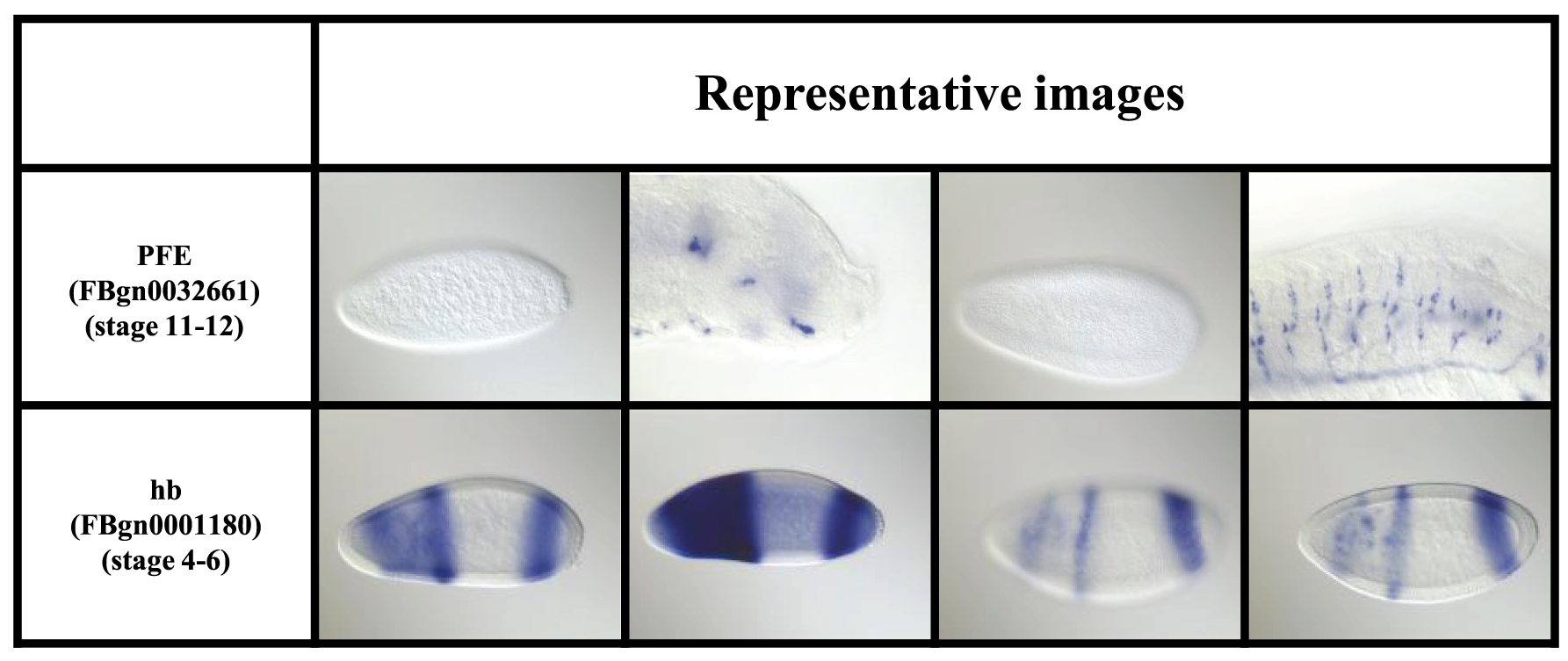

Supplement: Figure S5 — Complexity of the image data (focal distance and viewing angles). Two examples where several images corresponding to two individual genes (at stages 4–6 and 11–12, respectively) are either out of focus, with no visible gene expression pattern, or under tilted viewing angles, making the annotation process more difficult. (TIF) [file pcbi.1002098.s005.tif]

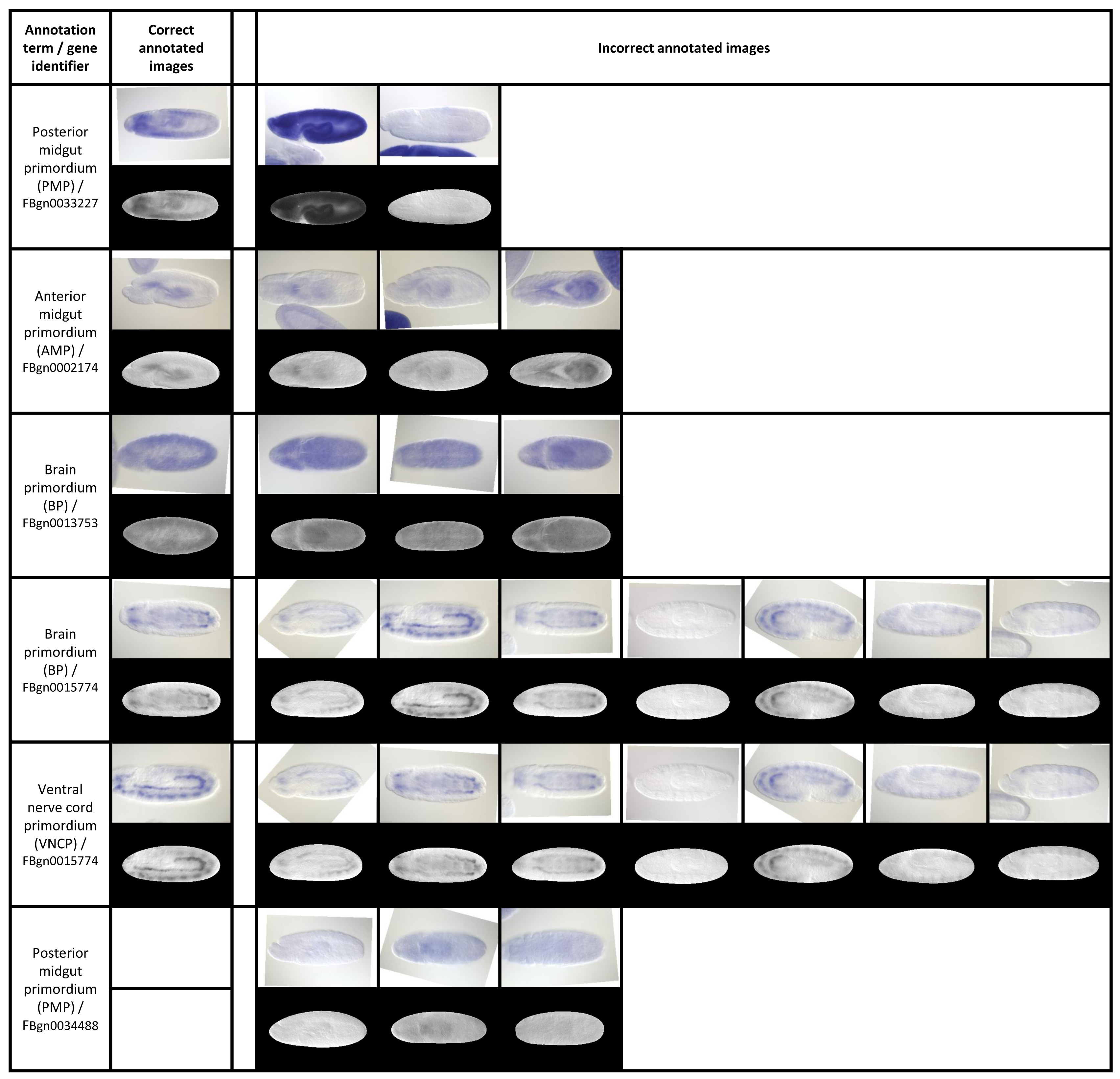

Supplement: Figure S6 — Spatial expression patterns of genes with successful classification within the minority voting scenario, data set . Several examples where, for a given gene, there is only one correct annotated image, due to out of focus, non-informative patterns or an improper rotation by the registration process. In each case, the original gene spatial expression patterns and the automatically extracted individual embryos are shown. (TIF) [file pcbi.1002098.s006.tif]

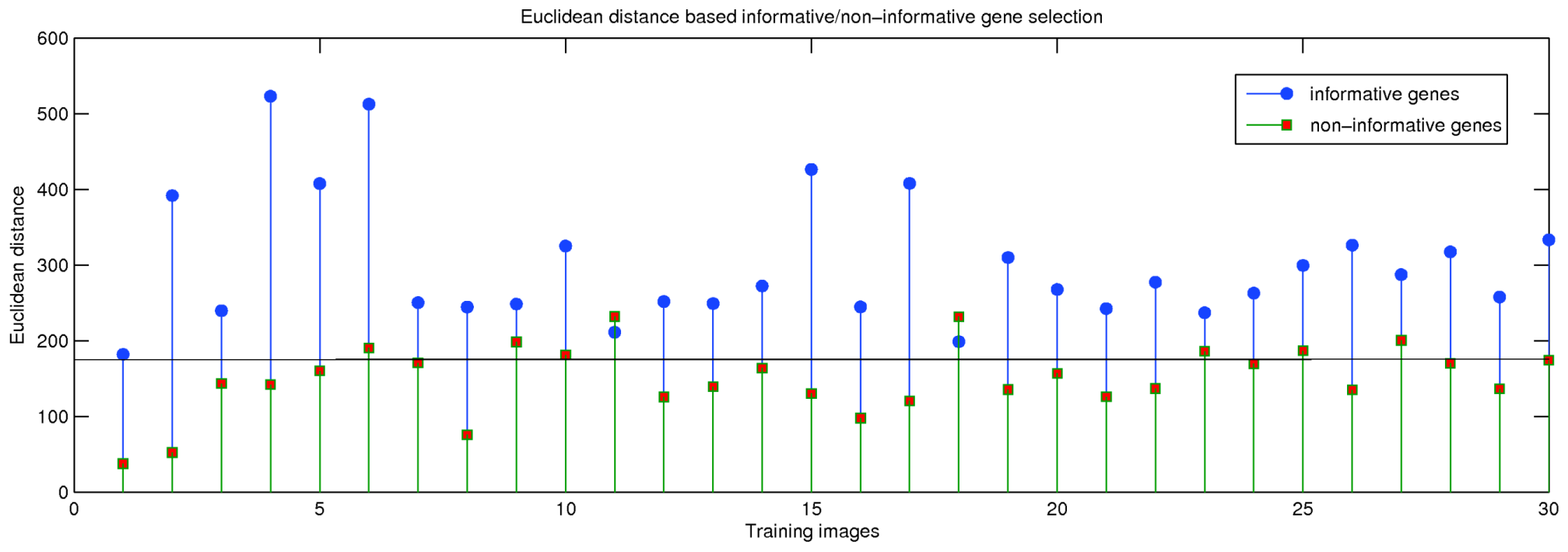

Supplement: Figure S7 — Euclidean distance based informative/non-informative gene selection. Informative images (lateral, dorsal/ventral expressions) are separated from non-informative images (mostly maternal expressions) through the use of Euclidean distance between their corresponding estimated mixing weights (rows in matrix ) and a reference vector. The chosen threshold is further employed to succesfully remove a total of non-informative images from data set . (TIF) [file pcbi.1002098.s007.tif]

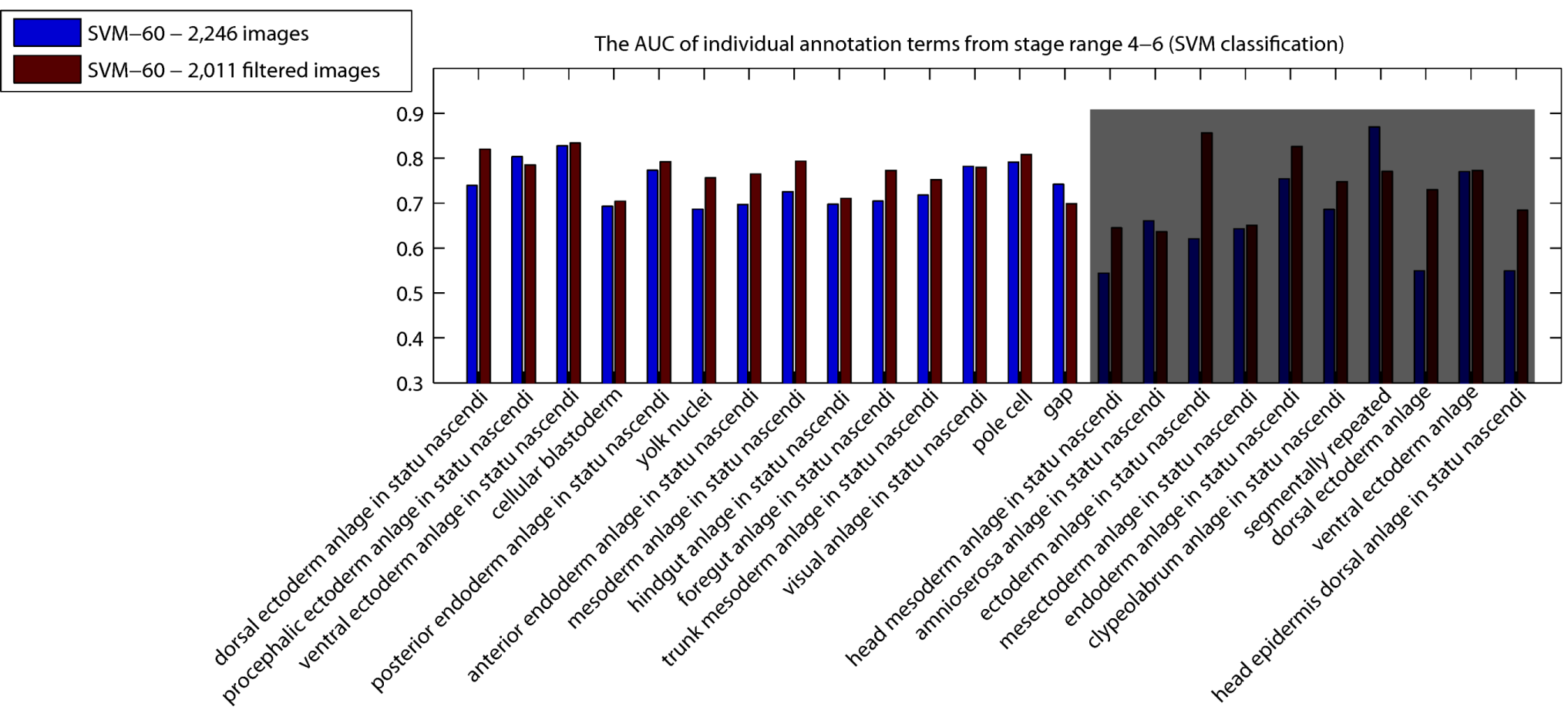

Supplement: Figure S8 — SVM analysis on developmental stages 4–6. The AUC values achieved by the SVM framework on the filtered data set ( images) in comparison to the AUC results on the original data set . (TIF) [file pcbi.1002098.s008.tif]

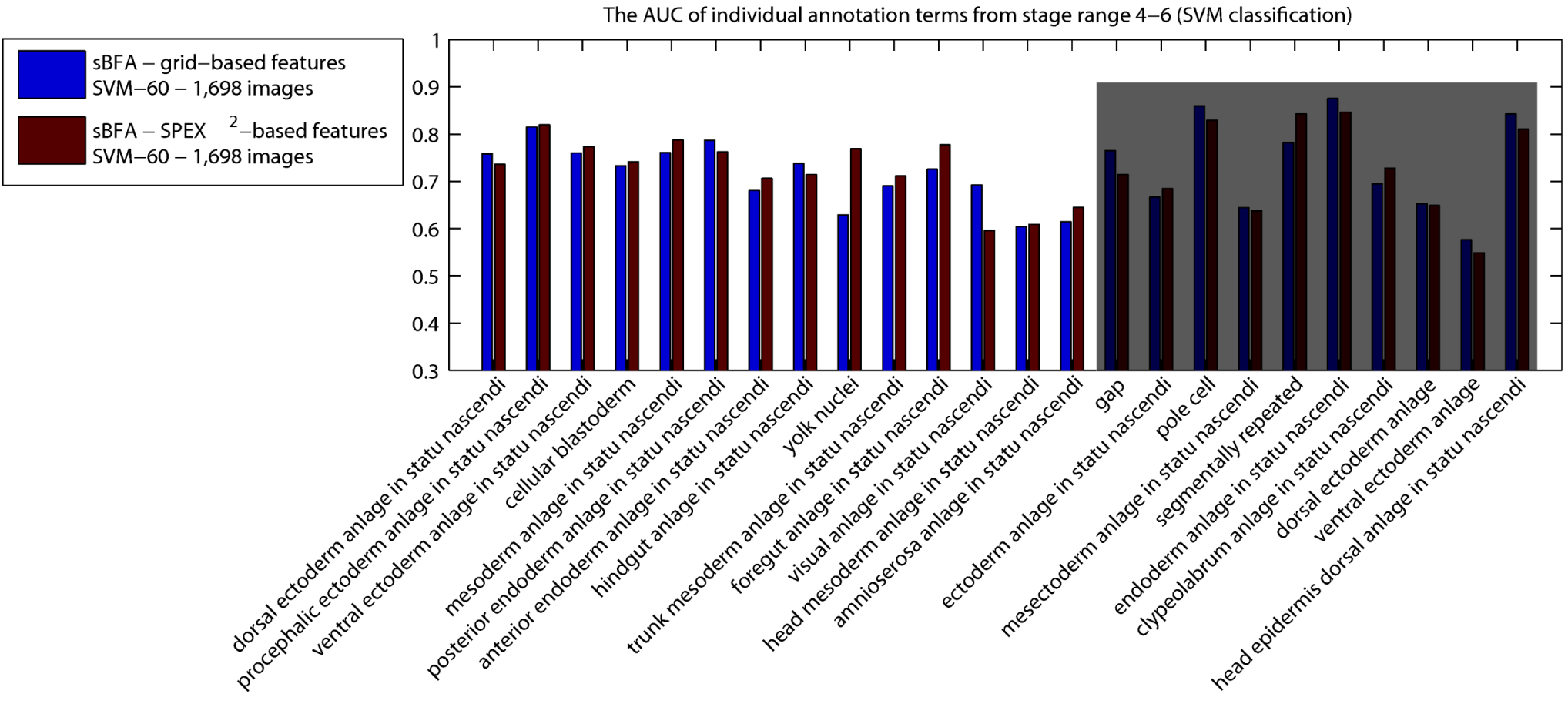

Supplement: Figure S9 — SVM analysis on developmental stages 4–6: the AUC of individual annotation terms using the sBFA model. We consider two different scenarios: using the grid-based features (grid size of ×), or using the -based features. The common set between the two studies extends to a total of images, with different views (lateral, dorsal/ventral). During the sBFA estimation, we used a number of factors , for both scenarios; the polynomial SVM generated the AUC of individual annotation terms. (TIF) [file pcbi.1002098.s009.tif]

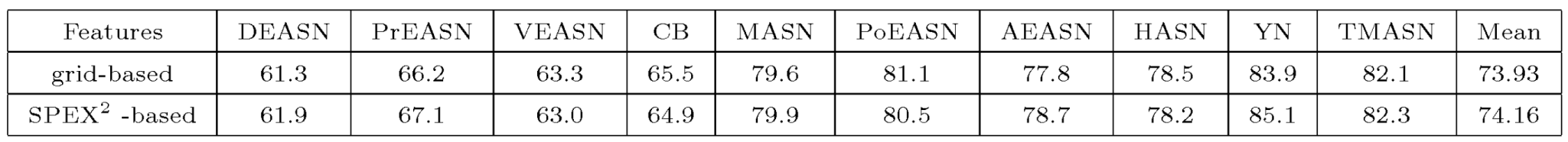

Supplement: Table S1 — Overall recognition rate () of the sBFA model on grid-based features and -based features, developmental stages . Image level recognition rates on the top most frequent annotation terms from the time window of developmental stage ( images), as used as metric in [30]. Abbreviations of the anatomical annotations: AEASN - anterior endoderm anlage in statu nascendi; CB - cellular blastoderm; DEASN - dorsal ectoderm anlage in statu nascendi; HASN - hindgut anlage in statu nascendi; MASN - mesoderm anlage in statu nascendi; PoEASN - posterior endoderm anlage in statu nascendi; PrEASN - procephalic ectoderm anlage in statu nascendi; TMASN - trunk mesoderm anlage in statu nascendi; VEASN - ventral ectoderm anlage in statu nascendi; YN - yolk nuclei. (TIF) [file pcbi.1002098.s010.tif]
